# Evaluating Different Virulence Traits of *Klebsiella pneumoniae* Using *Dictyostelium discoideum* and Zebrafish Larvae as Host Models

**DOI:** 10.3389/fcimb.2018.00030

**Published:** 2018-02-09

**Authors:** Andrés E. Marcoleta, Macarena A. Varas, Javiera Ortiz-Severín, Leonardo Vásquez, Camilo Berríos-Pastén, Andrea V. Sabag, Francisco P. Chávez, Miguel L. Allende, Carlos A. Santiviago, Octavio Monasterio, Rosalba Lagos

**Affiliations:** ^1^Laboratorio de Biología Estructural y Molecular, Departamento de Biología, Facultad de Ciencias, Universidad de Chile, Santiago, Chile; ^2^Laboratorio de Microbiología de Sistemas, Departamento de Biología, Facultad de Ciencias, Universidad de Chile, Santiago, Chile; ^3^Laboratorio de Microbiología, Departamento de Bioquímica y Biología Molecular, Facultad de Ciencias Químicas y Farmacéuticas, Universidad de Chile, Santiago, Chile; ^4^Departamento de Biología, Facultad de Ciencias, Centro FONDAP de Regulación del Genoma, Universidad de Chile, Santiago, Chile

**Keywords:** hypervirulent *Klebsiella pneumoniae*, intracellular survival, resistance to phagocytosis, host-pathogen interactions, *Danio rerio*, *Dictyostelium discoideum*

## Abstract

Multiresistant and invasive hypervirulent *Klebsiella pneumoniae* strains have become one of the most urgent bacterial pathogen threats. Recent analyses revealed a high genomic plasticity of this species, harboring a variety of mobile genetic elements associated with virulent strains, encoding proteins of unknown function whose possible role in pathogenesis have not been addressed. *K. pneumoniae* virulence has been studied mainly in animal models such as mice and pigs, however, practical, financial, ethical and methodological issues limit the use of mammal hosts. Consequently, the development of simple and cost-effective experimental approaches with alternative host models is needed. In this work we described the use of both, the social amoeba and professional phagocyte *Dictyostelium discoideum* and the fish *Danio rerio* (zebrafish) as surrogate host models to study *K. pneumoniae* virulence. We compared three *K. pneumoniae* clinical isolates evaluating their resistance to phagocytosis, intracellular survival, lethality, intestinal colonization, and innate immune cells recruitment. Optical transparency of both host models permitted studying the infective process *in vivo*, following the *Klebsiella*-host interactions through live-cell imaging. We demonstrated that *K. pneumoniae* RYC492, but not the multiresistant strains 700603 and BAA-1705, is virulent to both host models and elicits a strong immune response. Moreover, this strain showed a high resistance to phagocytosis by *D. discoideum*, an increased ability to form biofilms and a more prominent and irregular capsule. Besides, the strain 700603 showed the unique ability to replicate inside amoeba cells. Genomic comparison of the *K. pneumoniae* strains showed that the RYC492 strain has a higher overall content of virulence factors although no specific genes could be linked to its phagocytosis resistance, nor to the intracellular survival observed for the 700603 strain. Our results indicate that both zebrafish and *D. discoideum* are advantageous host models to study different traits of *K. pneumoniae* that are associated with virulence.

## Introduction

Bacterial pathogens are currently a major concern for public health, mainly due to the development of multiresistant strains, the tardy development of new effective antimicrobials or alternative methods to control infections, and the rising of hypervirluent phenotypes. Among them, the Gram-negative bacilli *Klebsiella pneumoniae* was declared by several international healthcare organizations as an “urgent threat to human health,” and represents the “K” of the “ESKAPE” pathogens, the six most significant and dangerous causes of hospital-acquired multiresistant infections (Centers for Disease Control and Prevention, U.S.A. 2013; UK Department of Health and Department for Environment Food and Rural Affairs, 2013). Additionally, although *K. pneumoniae* has been traditionally considered as an opportunistic pathogen causative of hospital-acquired infections, an increasing number of community-acquired invasive *K. pneumoniae* infections are being reported globally. They are presented mainly in the form of pyogenic liver abscesses that are often accompanied by severe metastatic infections such as meningitis, necrotizing fasciitis and osteomyelitis (Siu et al., 2012; Struve et al., 2015; Prokesch et al., 2016).

Several factors have been associated to *K. pneumoniae* pathogenesis, which contribute to enhance invasiveness, overcome host defenses, and resist antimicrobial chemotherapy (recently reviewed in Clegg and Murphy, 2016; Paczosa and Mecsas, 2016). Among them are the lipopolysaccharide and the O antigen, providing serum resistance; siderophores such as aerobactin and salmochelin, which scavenge iron in environments where it is scarce (as in mammal's fluids); type 1 and type 3 fimbriae involved in biofilm formation and tissue colonization; and a wide repertoire of extended-spectrum beta-lactamases and carbapenemases, conferring resistance to the majority of beta-lactams and carbapenems antibiotics. One of the most studied *K. pneumoniae* virulence determinant is the thick polysaccharide capsule that surrounds the organism (also called K antigen), which confers resistance to antimicrobial peptides, binds to and neutralizes K antigen-specific antibodies, and mediates resistance to phagocytosis by human neutrophils and macrophages (Simoons-Smit et al., 1986; Domenico et al., 1994; Campos et al., 2004). It also plays a role in biofilm formation (Balestrino et al., 2008), in establishing infection and activating host immune responses (Lawlor et al., 2005), and in facilitating intestinal colonization (Favre-Bonte et al., 1999). Non-capsulated *K. pneumoniae* strains are significantly less virulent than isogenic encapsulated strains in mouse models (Paczosa and Mecsas, 2016). Moreover, hypervirulent strains commonly have a hypermucoviscous phenotype, producing a thick capsule formed by a mucoviscous exopolysaccharide. Notably, K1 and K2 capsule types are the most associated with hypervirulent strains, and with an increased resistance to phagocytosis and intracellular killing by alveolar macrophages and neutrophils (Li et al., 2014). In addition, although *K. pneumoniae* is classically defined as an extracellular pathogen, it was recently reported that an isolate of this species could survive within macrophages in a vacuolar compartment, triggering apoptotic pathways and avoiding delivery to lysosomes (Cano et al., 2015). However, further studies must be performed to corroborate these findings, and to identify the bacterial determinants behind such intracellular survival. Moreover, additional studies are needed to understand the molecular basis of the hypervirulent phenotype.

The study of *K. pneumoniae* virulence has been performed classically in animal models such as mice, rats, and pigs (Clegg and Murphy, 2016; Paczosa and Mecsas, 2016). However, the use of mammalian hosts are limited by: (1) the need of costly facilities for their maintenance and manipulation, (2) the restricted possibilities for real-time analyses, (3) the need of invasive sample acquisition, and (4) ethical and practical restrictions. As an alternative, simple host models such as the wax moth *Galleria mellonella*, the worm *Caenorhabditis elegans*, the soil amoeba *Dictyostelium discoideum* and the fish *Danio rerio* (zebrafish) have been successfully employed for studying pathogenesis of different bacterial species, as well as in the screening for and testing of antimicrobial or anti-virulence drugs (reviewed in Bozzaro and Eichinger, 2011; Meijer and Spaink, 2011; Glavis-Bloom et al., 2012). Each of these phylogenetically distant organisms possess particular advantages and limitations as model hosts. Moreover, studying the interactions of a given pathogen with each of them provides largely complementary points of view of its virulence potential in different infection contexts and environmental conditions. Recent works used *G. mellonella* to characterize different *K. pneumoniae* virulence factors including Lipid A and capsular type determinants, as well as its interactions with the host immune system (Insua et al., 2013; Wand et al., 2013; Mills et al., 2017). Also, *G. mellonella* has been used to test the efficacy of antimicrobial agents against carbapenemase-producing strains of this species (McLaughlin et al., 2014; Wei et al., 2017). Similarly, *C. elegans* model have been employed to study the contribution of distinct factors such as efflux pumps and other outer membrane proteins on *K. pneumoniae* pathogenesis and antimicrobial resistance (Srinivasan et al., 2012; Lavigne et al., 2013; Bialek-Davenet et al., 2015). In this work, we further exploited the advantages of *D. discoideum* and zebrafish larvae as host models to develop new assays for studying *K. pneumoniae* pathogenesis.

*D. discoideum* is a soil-borne amoeba that feeds on environmental bacteria by phagocytosis. Therefore, it can be a natural host of opportunistic bacteria and thus has developed strategies to avoid invasion by given pathogens or to counteract their intracellular survival and replication (Cosson and Soldati, 2008; Clarke, 2010). As a professional phagocyte, it can be infected with different bacterial pathogens, and relevant virulence factors in mammals have been shown to be important in the interaction with this amoeba. Also, this organism is easy to grow and maintain in the laboratory, allowing the study of cellular processes with a variety of available genetic and biochemical tools. In addition, the existence of online resources like dictyBase permits easy access to genomic data and biological material such as plasmids and mutant strains (Kreppel et al., 2004; Basu et al., 2013; Fey et al., 2013). Consequently, this amoeba has been successfully used as a model to study virulence and the associated host response in different bacterial species, including *Pseudomonas* (Cosson et al., 2002; Bravo-Toncio et al., 2016), *Salmonella* (Sillo et al., 2011; Riquelme et al., 2016), *Legionella*, and *Mycobacterium* (reviewed in Steinert, 2011). Moreover, *D. discoideum* has been used to identify host factors required for sensing and intracellular killing of *K. pneumoniae* by phagocytic cells (Lima et al., 2014; Leiba et al., 2017). Also, it allowed investigating the role of *K. pneumoniae* surface structures on its interaction with phagocytes (March et al., 2013), and to identify factors associated with resistance to phagocytosis in the hypervirulent strain NTUH-K2044 (Pan et al., 2011). These studies support the relevance of exploring new assays to better exploit the *D. discoideum* model to investigate *Klebsiella*-host interactions.

Zebrafish (*Danio rerio*) is a vertebrate host model that harbors a mammalian-like innate immune system, and thus has been widely used for the study of host-pathogen interactions and the associated immune response (Kanther and Rawls, 2010). Transgenic zebrafish lines with fluorescently-labeled macrophages and other leukocyte populations allow non-invasive live-cell imaging during the optically transparent early life stages, thus enabling direct, real-time examination of the host response to infection (Renshaw et al., 2006). In previous work, we successfully used zebrafish larvae to study intestinal colonization and lethality by *Salmonella* Typhimurium, using two methods of infection: static immersion and microinjection (Varas et al., 2017a,b). In this regard, a recent work used adult zebrafish to test the safety and the efficacy *in vivo* of molecules with antibacterial activity against multiresistant *K. pneumoniae*, indicating that this pathogen can infect zebrafish when administered by intramuscular injection (Cheepurupalli et al., 2017). However, it remained unknown if *K. pneumoniae* can infect zebrafish in its larval stage, which harbors advantageous features compared to adult zebrafish as a host model, permitting live-cell imaging of the infection, an easier manipulation of the individuals, and larger population sizes that allow more robust statistical analyses.

Here, we exploited both the *D. discoideum* and zebrafish larvae host models to study virulence-related traits of three relevant clinical isolates of *K. pneumoniae*: ATCC 700603, ATCC BAA-1705, and RYC492. *Kp* 700603 is a multiresistant isolate obtained from urine at the Medical College of Virginia (Richmond, USA) in 1994 (Rasheed et al., 2000). It was the first *K. pneumoniae* strain identified that carries the SHV-18 extended-spectrum β-lactamase and it is used as a positive standard for antibiotic resistance mediated by this kind of enzymes. *Kp* BAA-1705 is another multiresistant strain isolated from urine in USA (2007), which is used as a standard for the production of KPC-2 carbapenemase. *Kp* RYC492 is a clinical isolate of fecal origin found in a screening of microcin-producing strains performed at the Microbiology Department at the Centro Especial Ramon y Cajal in Madrid (Asensio et al., 1976; de Lorenzo et al., 1984). This strain produces microcin E492 (MccE492), a bacteriocin with toxic activity against members of *Enterobacteriaceae*, antitumorigenic properties, and which forms amyloid fibers modulating its toxicity (Lagos et al., 2009; Marcoleta et al., 2013a). Determinants for production of MccE492 along with salmochelin-like siderophores are encoded in a genomic island found to be highly prevalent among hypervirulent *K. pneumoniae* isolates (Marcoleta et al., 2016). Our proposed assays using *D. discoideum* and zebrafish larvae as host models allowed us to evaluate and compare relevant virulence-related traits among these *K. pneumoniae* strains, including resistance to phagocytosis, intracellular survival, intestinal colonization, neutrophil recruitment and lethality. Complementation of these results with genomic and simple microbiological characterizations allowed us to observe striking differences in the virulence strategies between different strains of this species, pointing to the capsule as a major determinant for the pathogenesis in these surrogate models, as it was observed in mammalian host models.

## Materials and methods

### Bacterial strains, plasmids, and growth conditions

The bacterial strains and plasmids used in this work are listed in Table 1. *K. pneumoniae, K. aerogenes* and *E. coli* DH5α strains were routinely grown at 37°C with shaking (180 rpm) in Luria-Bertani broth (10 g/L triptone, 5 g/L yeast extract, 5 g/L NaCl) until late-exponential phase of growth. *K. pneumoniae* ATCC 700603 and ATCC BAA-1705 were kindly provided by Dr. Roberto Vidal from Facultad de Medicina, Universidad de Chile. For fluorescence labeling, bacterial strains were transformed by electroporation with pFCcGi, allowing the constitutive expression of the mCherry protein (Figueira et al., 2013; Addgene Plasmid #59324), or with pFCcGi-TMP. This last plasmid is a pFCcGi-derivative constructed by our group, where a dihydrofolate reductase gene expression cassette (conferring resistance to trimethoprim) was inserted in the *Pvu*I site, interrupting the beta-lactamase gene.

**Table 1:** Bacterial strains and plasmids used in this study.

**Bacterial strains or plasmids**	**Genotype or comments**	**Source/Reference**
*Klebsiella pneumoniae* RYC492	Produces microcin E492 and salmochelin siderophores, isolated from stool in Spain (1976). Resistant to kanamicin and ampicillin.	Laboratory collection (Asensio et al., 1976)
*Klebsiella pneumoniae* ATCC 700603	Isolated from urine in the USA (1994), multiresistant. Control for the production of extended spectrum β-lactamase SHV-18.	Roberto Vidal's laboratory collection. Also available from ATCC
*Klebsiella pneumoniae* ATCC BAA-1705	Isolated from urine in the USA (2007), multiresistant. Control for the production of KPC-2 carbapenemase.	Roberto Vidal's laboratory collection. Also available from ATCC
*Klebsiella aerogenes* DBS0305928	Recommended strain for supporting the growth of *D. discoideum*	Dicty Stock Center (Fey et al., 2007)
*Escherichia coli* DH5α	Laboratory strain typically used for cloning purposes.	Laboratory collection
pFCcGi	Constitutive expression of the mCherry protein, Amp^r^.	Figueira et al. (2013), retrieved from Addgene
pFCcGi-TMP	pFCcGi-derivative, where the β–lactamase gene was interrupted by a cassette conferring resistance to trimethoprim.	This work

### *Dictyostelium* strains and culture conditions

*D. discoideum* strains AX4 (DBS0302402) and AX2 *vatM*-GFP (DBS0235537) were obtained from Dicty Stock Center (Kreppel et al., 2004; Basu et al., 2013; Fey et al., 2013), and cultured according to standard protocols (Fey et al., 2007). Briefly, *D. discoideum* strains were maintained at 23°C in SM medium (10 g/L glucose, 10 g/L peptone, 1 g/L yeast extract, 1 g/L MgSO_4_ × 7H_2_O, 1.9 g/L KH_2_PO_4_, 0.6 g/L K_2_HPO_4_, 20 g/L agar), growing on a confluent lawn of *Klebsiella aerogenes* DBS0305928. Before the assays, amoebae were grown at 23°C with agitation (180 rpm) in liquid HL5 medium (14 g/L tryptone, 7 g/L yeast extract, 0,35 g/L Na_2_HPO_4_, 1,2 g/L KH_2_PO_4_, 14 g/L glucose, pH 6,3) in the absence of bacteria (axenic cultures). When required, media were supplemented with streptomycin (300 mg/L), ampicillin (100 mg/L) or G418 (geneticin, 10 mg/L). Amoebae were harvested in early exponential phase (1–2 × 10^6^ cells/mL) and centrifuged at 500 × g for 5 min. Prior to the infection assays, the supernatant was discarded and the pellet was washed three times using Soerensen buffer (2 g/L KH_2_PO_4_, 0.36 g/L Na_2_HPO_4_ × 2H_2_O, pH 6.0) or adjusted to 5 × 10^7^ cells/mL in HL5 medium for the social development assays. Determination of viable amoeba cells was performed by Trypan blue exclusion and counting in a Neubauer chamber.

### *D. discoideum* social development assays

For social development assays, overnight cultures of each bacterial strain (30 μL) were homogeneously included per well of a 24-well plate containing N agar (1 g peptone, 1 g glucose, 20 g agar in 1 L of 17 mM Soerensen phosphate buffer) (Sillo et al., 2011) and grown overnight at 23°C. A drop of a cellular suspension corresponding to 5 × 10^5^ *D. discoideum* AX4 cells in HL5 was spotted in the middle of each well and the plates further incubated at 23°C for 5 days. The social development of amoebae was monitored daily during 5 days and the phase reached was scored, being classified as “aggregation,” “elevation” and “culmination.” A score of “1” was assigned when amoebae aggregated forming a phagocytosis plate, “2” when across all the well surface elevated structures such as worms or fingers were observed, and “3” when fruiting bodies were formed across all the well surface. Transitions among any of these three phases were scored with half of the value corresponding to the closest next stage. Additionally, images of social development were obtained at days 1, 2, and 3 by using an Olympus MVX10 stereomicroscope with a total magnification of 20X.

### Intracelullar survival assays

*D. discoideum* AX2 *vatM*-GFP grown axenically (~1 × 10^7^ cells) was co-incubated with each bacterial strain at 23°C with agitation (180 rpm) in 5 mL of Soerensen buffer using a multiplicity of infection (MOI) of 50 bacteria/amoeba. To evaluate the intracellular survival, amoebae were washed three times with Soerensen buffer to remove extracellular bacteria after 1 h of co-incubation. Then, infected cells were suspended in 5 mL of Soerensen buffer (*t* = 0) and further incubated at 23°C with agitation. Aliquots were obtained at 0, 1.5, 3, 6, and 24 h post-infection. Viable amoebae were determined at each time point. In parallel, infected amoebae recovered at each time point were treated for 10 min with 20 μg/mL gentamycin to remove extracellular bacteria, washed with Soerensen buffer, and lysed with 0.2% Triton X-100. Titers of intracellular bacteria were determined by serial dilutions and plating on LB agar. Statistical significance was determined using a one-way ANOVA and two-way ANOVA with a Fisher's LSD post-test, as described previously (Riquelme et al., 2016). Also, aliquots of the mixed cultures were mounted for laser-scanning confocal microscopy analysis.

### Resistance to phagocytosis assays

To quantify extracellular bacteria, *D. discoideum* AX2 *vatM*-GFP (1 × 10^7^ cells) and mCherry-labeled bacteria were co-incubated at 23°C for 24 h in 5 mL of Soerensen buffer using a MOI of 100 bacteria/amoeba. Aliquots were obtained at 0, 3, and 24 h post-infection. Amoebae were removed by centrifugation at 500 × g for 5 min and extracellular bacteria were determined at each point by serial dilutions and plating on LB agar. Statistical significance was determined using a two-way ANOVA with Fisher's LSD post-test. In parallel, aliquots of the mixed cultures were extracted and mounted for laser-scanning confocal microscopy analysis.

### Laser scanning confocal microscopy

Images of infected amoeba cells from phagocytosis assays were acquired using a Zeiss LSM 700 laser scanning confocal microscope equipped with an Alpha Plan Fluar 100X/1.45 Oil M27 optic setup. Prior to observation, cells were mounted on a thin layer of 1.5% agarose in PBS buffer and deposited on a glass slide. To visualize GFP-associated fluorescence (amoebae), the sample was excited at 488 nm with an argon laser and emission was detected using a filter in the 493–549 nm range. To visualize mCherry-associated fluorescence (bacteria), the sample was excited at 543 nm with a HeNe laser and emission was detected using a filter in the 548–679 nm range. Images were acquired using the ZEN 2010 software (Zeiss), and analyzed using Fiji and ImageJ softwares (Schindelin et al., 2012; Schneider et al., 2012), as described previously (Riquelme et al., 2016). Microphotographs of the phagocytosis assays are presented with a total magnification of 550X. Images of capsule-stained *K. pneumoniae* were obtained using a Zeiss LSM 700 laser scanning confocal microscope equipped with an Alpha Plan Fluar 100X/1.45 Oil M27 optic setup, and a laser-mediated bright field mode. Microphotographs of stained *K. pneumoniae* cells are presented with a total magnification of 3000X (**Figure 6**) or of 2000X (Supplementary Figure 4).

### Zebrafish husbandry

Zebrafish (*Danio rerio*) embryos were obtained by natural spawning of Tab5 (wild type), Tg(*BACmpo:gfp*) (Renshaw et al., 2006) and Tg(*fli1:egfp*) lines (Lawson and Weinstein, 2002). Fertilized eggs were raised in Petri dishes containing E3 medium (5 mM NaCl, 0.17 mM KCl, 0.33 mM CaCl_2_, 0.3 mM MgSO4) and 0.1% methylene blue until 3 days post-fertilization (dpf). All procedures complied with national guidelines of the Animal Use Ethics Committee of the University of Chile and the Bioethics Advisory Committee of FONDECYT-CONICYT (the national funding agency for this work).

### Bacterial injection experiments

Bacterial injection experiments were adapted from previously described methods to evaluate *S*. Typhimurium virulence (Díaz-Pascual et al., 2017; Varas et al., 2017a,b). For the injection assays, the bacterial cultures were washed and subsequently suspended in phosphate-buffered saline (PBS) and concentrated 5-fold (*K. pneumoniae* strains) or 7.5-fold (*E. coli* DH5α). Zebrafish larvae (3-dpf) were anesthetized with 4.2% tricaine and mounted on 1% low melting point agarose. The zebrafish were kept under anesthesia during the whole injection procedure. 1–5 nL (15,000–18,000 CFU) were injected in the circulatory system via the caudal artery (to evaluate survival), or in the otic vesicle (to evaluate neutrophil recruitment and bacterial burden). To determine the CFU injected, the droplets were also diluted in sterile PBS and plated on LB-agar. Infected larvae were transferred into 6-well plates containing fresh E3 medium and kept at 28°C for 72 h. Death events were quantified every 24 h, and survival curves were constructed and analyzed by means of the Kaplan-Meier method using the GraphPad Prism 6.0 software. Survival experiments were performed in triplicate for a total of 57 larvae per condition, while neutrophils recruitment experiments were done in triplicate for a total of 21 larvae per condition.

### Inflammation mediated by neutrophils and bacterial burden

Recruitment of neutrophils to the otic vesicle was observed using the Tg(*BACmpo:gfp*) zebrafish line. The larvae were observed using an Olympus MVX10 fluorescence stereomicroscope at 24 h post-injection (hpi). To measure neutrophil recruitment and the bacterial burden, injected zebrafish larvae were anesthetized with 0.1% tricaine and mounted on 1% low melting point agarose to photograph the injected area. Subsequently, each image was processed by quantifying the fluorescence intensity from the red channel (mCherry-labeled bacteria) and green channel (EGFP-labeled neutrophils) (ImageJ software). The region used to quantify the fluorescence was defined as the total area corresponding to the otic vesicle, which can be easily distinguished from bright field images of the larvae. In our conditions, this corresponded to a circular area of 550 × 550 pixels, which was the same for all the individuals evaluated. Microphotographs of the otic vesicle are presented with a total magnification of 40X. Statistical differences were assessed by means of a Kruskal-Wallis analysis followed by Dunn's multiple comparisons test using the GraphPad Prism 6.0 software.

### Bacterial immersion experiments

Immersion assays for *K. pneumoniae* were adapted from similar methods performed previously to study *S*. Typhimurium virulence (Díaz-Pascual et al., 2017; Varas et al., 2017a,b). Bacterial cultures were grown until exponential phase (OD_600_ = 0.4–0.6) and the cells were harvested by centrifugation, washed, and subsequently suspended in sterilized E3 medium. To determine the viable bacteria (CFU/mL), the suspension was serially diluted and plated on LB-agar. Zebrafish larvae (3-dpf) were washed with E3 medium and groups of 10 larvae were placed per well in a sterile 6-well plate. The wells were filled up to 8 mL with bacteria (5–8 × 10^8^ CFU/mL) and zebrafish larvae were incubated in this suspension at 28°C for 48 h. After the co-incubation, the larvae were washed with E3 medium and placed in a sterile 6-well plate filled with fresh E3 medium. The progression of the infection was monitored until 96 h post-exposition (hpe). Immersion experiments were performed in a total of 6 independent replicates, using a total of 75 larvae per bacterial strain. The progression of infection and bacterial colonization was monitored through live-cell imaging using an Olympus MVX10 fluorescence stereomicroscope at 48, 72, and 96 hpe. Photographs of the larvae are presented with a total magnification of 12X. To evaluate intestinal colonization, the gastrointestinal tract of the larvae was divided arbitrarily into three segments: anterior, middle and posterior. Four qualitative degrees of intestinal colonization were established based on the fluorescent signal observed under stereomicroscope: (0) no colonization, (1) low colonization, (2) intermediate colonization, and (3) high colonization. The total intestinal colonization score of each individual was calculated as the sum of the scores observed in each segment of the intestine, in a scale ranging from 0 to 9.

### Capsule staining

Capsule staining was performed as described previously (Breakwell et al., 2009). For this, cultures of each bacterial strain were grown overnight at 37°C without shaking. An aliquot of the culture was mixed with a drop of India ink (Mars® matic, Staedtler) and smeared over a glass slide, letting it to air-dry. Then, the smear was covered with a drop of crystal violet during 1 min, washed carefully with distilled water, and let to air-dry. The stained samples were observed by laser-scanning confocal microscopy using a 543 nm HeNe laser-mediated bright field mode.

### Biofilm formation assessment

Biofilm formation in abiotic surfaces was measured as described previously for *K. pneumoniae* (Bruchmann et al., 2015). Briefly, bacterial cultures were grown overnight at 37°C with shaking (180 rpm), and the cells were washed and suspended in fresh LB medium to an OD_600_ of 0.2. Wells of a sterile 96-well microtiter plate were inoculated with 100 μL of the bacterial suspensions, and incubated at 37°C without shaking. After 24 h, the wells were washed three times with 200 μL of sterile distilled water and stained with 150 μL of a 0.1% crystal violet aqueous solution for 30 min. Then, wells were washed three times with 200 μL of distilled water, crystal violet was extracted adding 200 μL of 95% ethanol and incubating for 30 min. After incubation, the absorbance at 590 nm was measured.

### Genomic analyses

The whole-genome sequences of the strains *K. pneumoniae* RYC492, ATCC 700603, and ATCC BAA-1705 were retrieved from the NCBI database, under the accession numbers APGM01000001.1, CP014696.2, and GCA_000349265.2, respectively. Genomic islands identification was performed through sequence analysis and multiple alignment using the platforms Artemis, ProgressiveMauve, and NCBI BLAST, as described previously (Marcoleta et al., 2016). The identification of capsule synthesis *loci* was performed using Kaptive (Wyres et al., 2016) and Kleborate (Lam et al., in review). The identification of genes related to yersiniabactin, colibactin, salmochelin and other siderophore production systems was done used the tool Kleborate. For the identification of fimbriae-related genetic clusters, all the known *K. pneumoniae* proteins participating in fimbriae biogenesis were retrieved from the UniProtKB database and were used as query to search for homologous proteins in each of the analyzed genome, using the NCBI BLAST tools. Identification of virulence factors using whole genome data was performed using the tool PathogenFinder (Cosentino et al., 2013).

## Results

Based in the use of *D. discoideum* and zebrafish larvae, we set up, optimized, and employed a repertoire of assays to study and compare a set of virulence-associated traits of three relevant *K. pneumoniae* clinical isolates. Two of them, ATCC 700603 and ATCC BAA-1705, correspond to the standard *K. pneumoniae* multiresistant strains producing the KPC-2 carbapenemase and the SHV-18 extended-spectrum β-lactamase, respectively. The third strain, RYC492, carries a genomic island found to be highly prevalent among hypervirulent *K. pneumoniae* isolates (Marcoleta et al., 2016). Although all three described strains have sequenced genomes (Broberg et al., 2013; Marcoleta et al., 2013b; Elliott et al., 2016), to our knowledge no previous studies assessed experimentally their virulence potential.

### Using *D. discoideum* social development cycle to evaluate *K. pneumoniae* virulence

**Figure 1:**
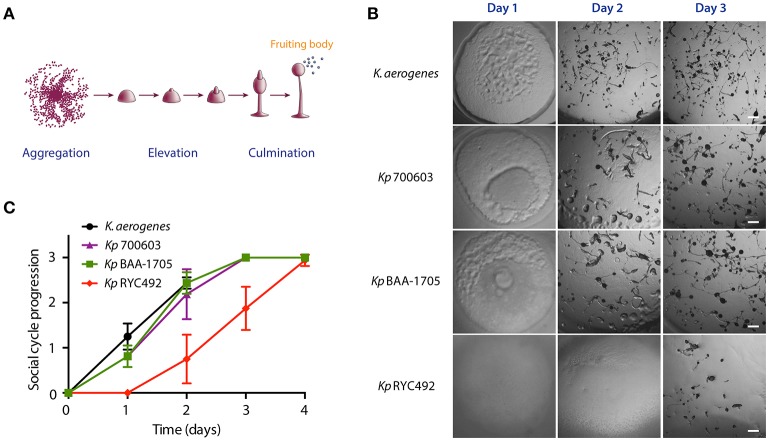
Use of the *D. discoideum* social development assay to evaluate *K. pneumoniae* virulence. **(A)** Main stages of the *D. discoideum* social cycle, which culminates with the formation of the fruiting bodies. The scheme was adapted from Fey et al. (2007). **(B)** Micrographs of the social cycle progression when feeding amoeba with different *K. pneumoniae* strains or with *K. aerogenes* DBS0305928 (control). Scale bar, 100 μm. **(C)** Semi-quantitative assessment of the social cycle progression in presence of different *Klebsiella* strains using several replicates. The symbols represent the mean of six independent experiments and the error bars show the standard deviation. The details on scoring criteria can be found in the Materials and Methods section.

As a first approach to exploit *D. discoideum*-*Klebsiella* interactions in the study of *K. pneumoniae* virulence, we adapted a social development assay previously described in a screening for *Pseudomonas aueruginosa* antivirulence compounds (Bravo-Toncio et al., 2016). This assay is based on that virulent bacteria delay the completion of *D. discoideum* social development, while attenuated or non-pathogenic bacteria allows its rapid conclusion (normally within 2–3 days in our experimental conditions). Thus, assessing the effect of a given bacterial strain over the social development is a way to estimate its virulence. Consequently, we compared the effect of feeding *D. discoideum* with the *K. pneumoniae* strains RYC492, 700603 and BAA-1705 over the social development of the amoeba. Additionally, *Klebsiella aerogenes* DBS0305928 was included as non-pathogenic control, since this strain is routinely used as a food to support *D. discoideum* growth in laboratory conditions (Fey et al., 2007). To this end, 24-well plates containing N agar were inoculated with the different strains and incubated overnight at 23°C to allow the growth of a thin bacterial lawn, which was of a similar prominence in all the strains tested (Supplementary Figure 1A). Moreover, the three *K. pneumoniae* strains showed a similar growth in N broth at 23°C (Supplementary Figure 1B), indicating that a comparable number of bacterial cells are present in the lawn formed by each strain before inoculating amoeba cells. Then, ~1 × 10^6^ *D. discoideum* cells were deposited over the bacterial lawns and incubated at 23°C for several days, monitoring the progress of the social development, which involves mainly three consecutive stages: aggregation, elevation and culmination (Figure 1A). The completion of the cycle is characterized by the formation of multiple spore-containing fruiting bodies, such as those observed normally after 2–3 days when amoebae were fed with *K. aerogenes* DBS0305928 (Figures 1B,C). At day 1, amoebae fed with *K. aerogenes* showed a complete phagocytosis plaque (where the whole bacterial lawn inside was engulfed), with aggregating cells forming early multicellular forms in the center. In contrast, incomplete phagocytosis plaques were observed after feeding amoebae with *K. pneumoniae* 700603 and BAA-1705 (Figure 1B). However, in the following days, no differences in the cycle progression were observed between these strains and the control (culminating between 2 and 3 days), indicating that *Kp* 700603 and *Kp* BAA-1705 are mildly virulent for *D. discoideum*. Conversely, *Kp* RYC492 showed a highly virulent phenotype causing a delay in the cycle, where at day 1 no clear phagocytosis plaque was observed, and the early elevation phase was reached after 3 days of co-incubation. Noteworthy, the fruiting bodies and earlier multicellular structures formed by amoebae fed with any of the *K. pneumoniae* strains tested showed an abnormal morphology. We observed mainly structures with an increased size, such as thicker stems and a bigger sorus, compared with that observed when fed with *K. aerogenes*. This suggests that *K. pneumoniae* somehow affects *D. discoideum* multicellular morphological plan, and that this could be further exploited as an additional read-out in this kind of virulence assays. Taken together, these results indicate that social development assays are a useful and straightforward way to compare virulence among distinct *K. pneumoniae* strains.

### Intracellular survival of *K. pneumoniae* in *D. discoideum*

**Figure 2:**
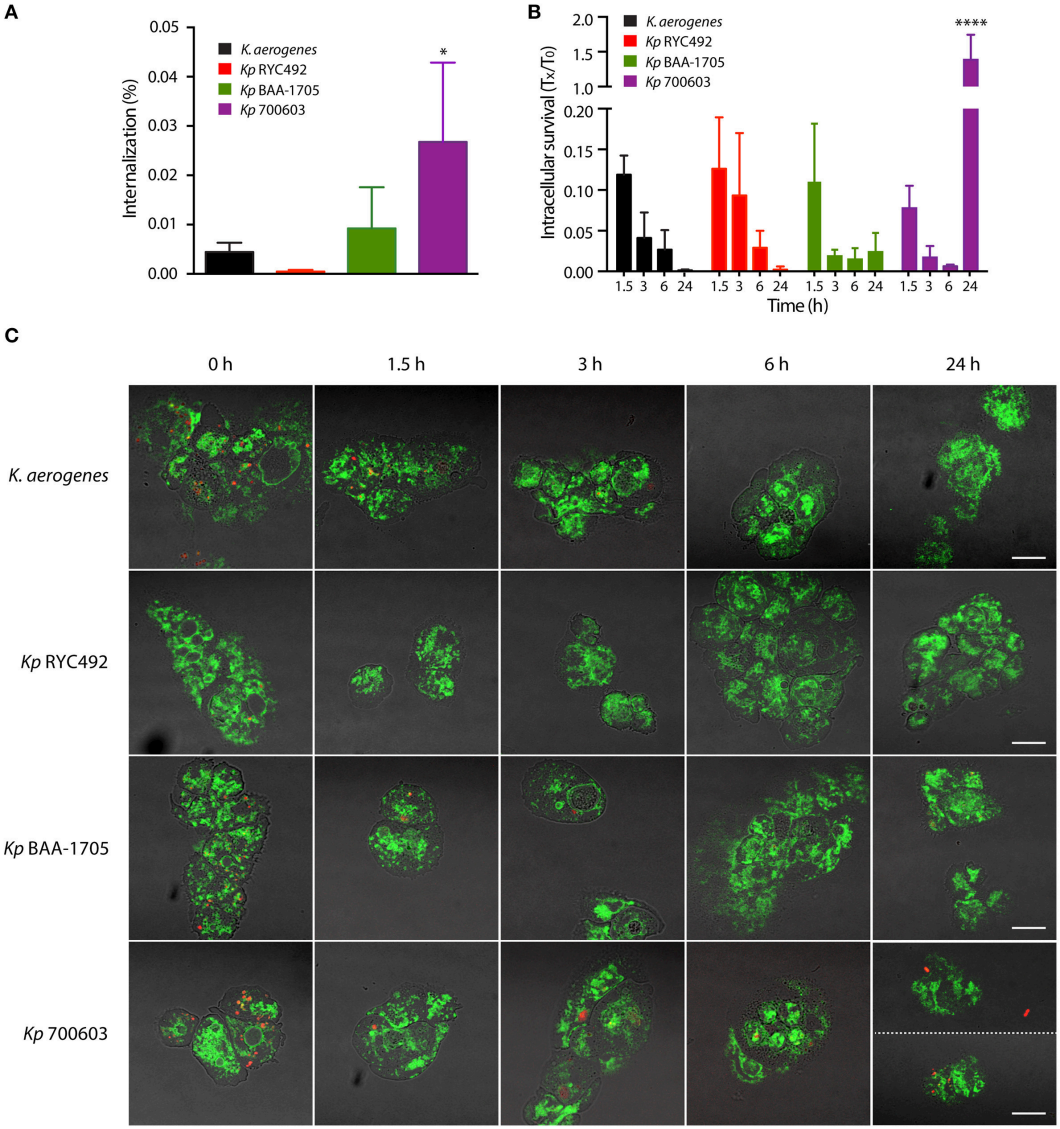
Bacterial phagocytosis assays revealed differences in invasiveness and intracellular survival among different *K. pneumoniae* strains. **(A)** Percentage of bacteria that were internalized after co-incubation of amoebae-*Klebsiella* for 1 h. **(B)** Total intracellular bacteria were titrated at the indicated times (T_x_), and the results were expressed as the fraction respect the survival at time zero (T_0_). In both **(A,B)**, the symbols represent the mean of three independent experiments and the standard deviation. ^*^p < 0.05, ^****^p < 0.0001. **(C)** Live-cell imaging of *D. discoideum* cells (green) interacting with *Klebsiella* (red), representative of the experiments showed in **(A,B)**. Bright field and fluorescence images were blended in order to better appreciate the boundaries of the amoeba cells. At time 24 h, two pictures of amoebae infected with *Kp* 700603 are shown in order to illustrate that both extracellular (upper picture) and intracellular bacteria (lower picture) could be observed. Scale bar, 10 μm.

As described previously, a major determinant of *K. pneumoniae* virulence is its capacity to evade or resist macrophage-mediated phagocytosis and killing. Previous work showed that assays using axenic cultures of *D. discoideum* can be used to study invasiveness and intracellular survival in *Salmonella* Typhimurium (*S*Tm) (Riquelme et al., 2016). Remarkably, null mutants in genes directly linked to *Salmonella* virulence (i.e., demonstrated to be attenuated in murine models) also showed an impaired ability to invade or to survive intracellularly in *D. discoideum*, indicating that *S*Tm (and probably other bacteria) uses a common set of genes and molecular mechanisms to infect amoeba and animal host cells. Thus, we adapted the previously described assays using vegetative *D. discoideum* cells, to evaluate the invasiveness and intracellular survival of *K. pneumoniae* strains. For this purpose, we co-incubated both organisms during 1 h to let amoebae feed on bacteria. Then, amoeba cells were washed to remove most of bacteria from the medium, and incubated at 23°C. At different time points, aliquots were extracted and treated with gentamycin to kill extracellular bacteria. Then, *D. discoideum* cells were lysed with Triton X-100, and intracellular bacteria recovered from infected amoebae were titrated. As a control, the same procedure was performed using *K. aerogenes* DBS0305928. Measuring the amount of bacteria that infected amoebae after 1 h of co-incubation (time 0 of the assay), we observed that the different strains were internalized at distinct levels (Figure 2A). While *Kp* 700603 was internalized significantly more than the control, *Kp* RYC492 internalization was considerably lower. Following viable intracellular bacteria over time, we observed that titers of all strains declined during the first 6 h, being almost undetectable after 24 h in the case of *Kp* RYC492 and *K. aerogenes* DBS0305928 (Figure 2B). Conversely, *Kp* 700603 survived and even replicated inside the amoebae after 24 h, showing a ~200-fold increase in the viable cell count between 6 and 24 h post-infection. Meanwhile, *Kp* BAA-1705 was internalized at similar levels than the control, and a small population of bacteria was detected after 24 h.

To further support our observations, we followed the fate of intracellular *K. pneumoniae* through live-cell imaging using laser-scanning confocal microscopy. To this end, a similar experiment was performed using *D. discoideum* cells expressing a calnexin-GFP protein fusion (Müller-Taubenberger et al., 2001), along with bacteria constitutively expressing the red fluorescent protein mCherry from plasmid pFCcGi-TMP. The interaction of intracellular bacteria and amoebae was monitored at 0, 1.5, 3, 6, and 24 h post-infection (Figure 2C, Supplementary Figure 2). In agreement with the bacterial count, a large number of red fluorescent bacteria were detected within amoeba after 1 h co-incubation with *Kp* 700603, *Kp* BAA-1705, and *K. aerogenes* DBS0305928, indicating that all these strains were rapidly internalized by *D. discoideum*. In the case of *Kp* RYC492, a notably smaller number of bacteria was detected at this time, which correlated with the low percentage of internalization measured for this strain (Figure 2A). In all the following time points, very few or no *Kp* RYC492 cells were detected in association with amoebae. Intracellular *Kp* BAA-1705 and *K. aerogenes* DBS0305928 were observed only until 3 h after the removal of the extracellular bacteria. In contrast, intracellular *Kp 700603* cells were observed at every time point evaluated. In fact, we were able to detect bacteria within *D. discoideum* after 24 h of co-incubation. These observations confirmed that *Kp* 700603 is able to survive and replicate intracellularly in *D. discoideum*. Additionally, some extracellular *Kp* 700603 were typically detected after 24 h post-infection, suggesting that this strain is able to escape from *D. discoideum* cells upon replication.

### Use of *D. discoideum* to evaluate *K. pneumoniae* resistance to phagocytosis

**Figure 3:**
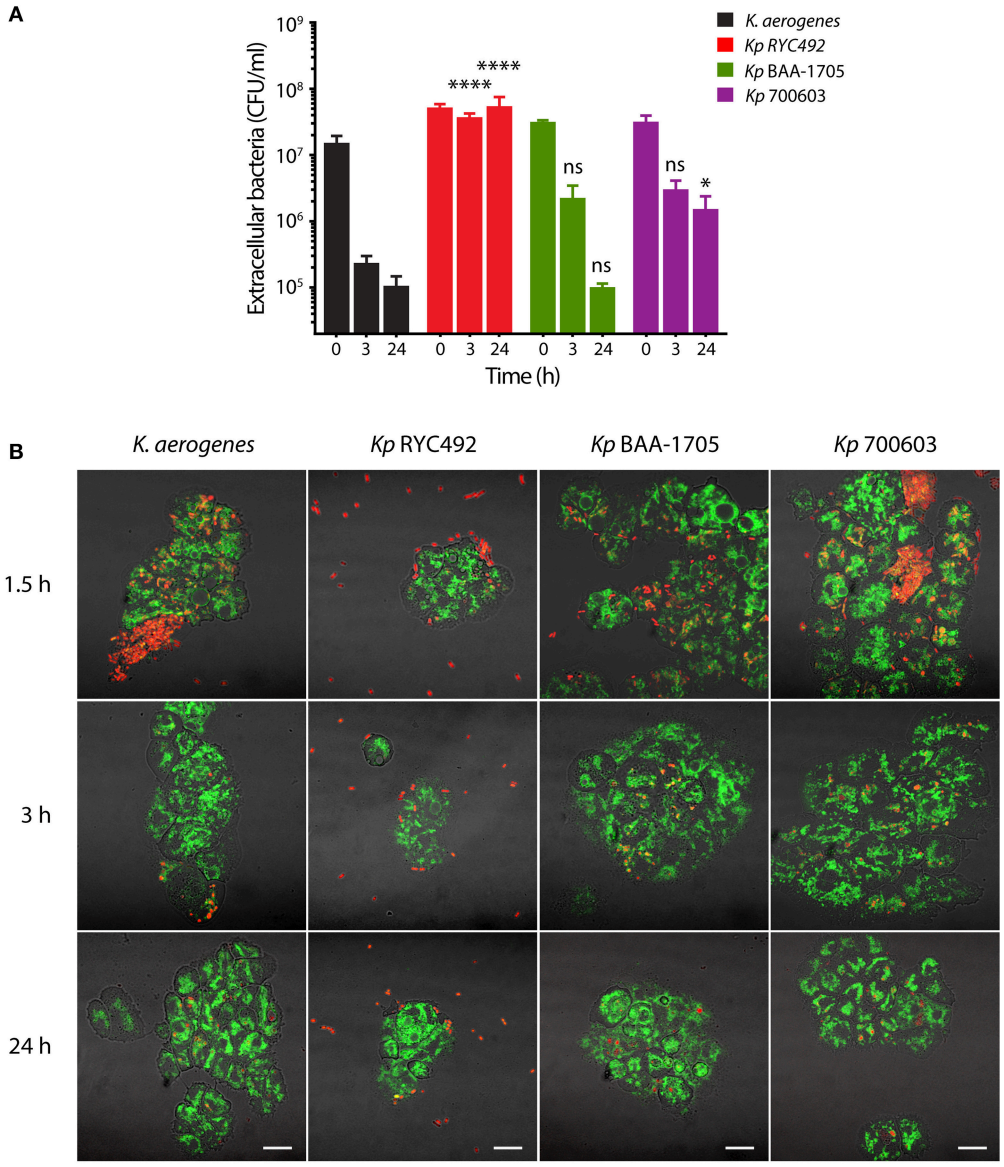
Evaluation of the resistance to phagocytosis of *K. pneumoniae* using *D. discoideum* as host cell model. **(A)** Extracellular viable bacteria count after different times of *Klebsiella*-amoeba co-incubation. At each indicated time, the mixed cultures were centrifuged at low rpm in order to sediment *D. discoideum* cells and intracellular bacteria, and the CFU remaining in the supernatant were titrated. The symbols represent the mean of three independent experiments and the error bars correspond to the standard deviation. At times 3 and 24 h, *Kp* RYC492 and *Kp* 700603 showed a significantly higher number of extracellular bacteria, compared with *K. aerogenes* (Two-way ANOVA, ^*^p < 0.05, ^****^p < 0.0001). **(B)** Confocal microscopy images representative of the experiment showed in **(A)**. At each indicated time, an aliquot of the mixed cultures was directly mounted for microscopic observation (without centrifuging). *D. discoideum* cells are green and bacterial cells are red. Bright field and fluorescence images were blended in order to better appreciate the boundaries of the amoeba cells. Scale bar, 10 μm.

The small proportion of *Kp* RYC492 cells that were internalized by *D. discoideum*, as well as the notably fewer bacteria found inside amoeba cells observed by confocal microscopy during the infection assays, suggested that this strain could avoid or prevent phagocytosis. To further investigate this possibility, we adapted the infection assay using axenic *D. discoideum* cultures described above. In this version, we allowed amoebae and bacteria to interact during the whole 24 h-period monitored. At distinct times, aliquots of the mixed cultures were obtained and mounted for microscopic observation, and the viable extracellular bacteria were titrated. This was performed centrifuging the aliquots at low speed to sediment *D. discoideum* cells along with intracellular bacteria, and then diluting and plating the supernatant. Although with different kinetics, phagocytic activity of *D. discoideum* caused the reduction over time in the viable count of all the tested bacterial strains except for *Kp* RYC492, which remained constant (Figure 3A). As expected, *D. discoideum* rapidly phagocytized *K. aerogenes* DBS0305928. On the other hand, *Kp* BAA-1705 and *Kp* 700603 extracellular bacteria showed a similar reduction upon 3 h of co-incubation with amoebae, but roughly 10-fold more bacteria of the *Kp* 700603 strain were observed after 24 h. This could be attributable to the ability of *Kp* 700603 to replicate inside *D. discoideum* cells. In accordance with the viable cell counts, observation of the mixed cultures by confocal microscopy revealed the presence of a high number of *Kp* RYC492 bacteria that were not associated with amoeba cells during all the times monitored (Figure 3B, Supplementary Figure 3). Conversely, for all the other strains most bacteria were found associated with *D. discoideum* cells, suggesting that *Kp* RYC492 not only avoid phagocytosis but also the adhesion to phagocytic cells. Taken together, these results show that *D. discoideum* is a suitable model to study different aspects of the *K. pneumoniae* virulence. In particular, they allowed uncovering striking differences among strains of this species, in the context of their interaction with the host, including traits widely related to pathogenesis such as resistance to phagocytosis, invasion and intracellular survival.

### Using zebrafish larvae as a host model for studying *K. pneumoniae* pathogenesis

Zebrafish larvae have shown to be a useful surrogate model to study the infective process of enteric bacterial pathogens (van der Sar et al., 2003; Clatworthy et al., 2009; Nguyen-Chi et al., 2014). To our knowledge, no studies have evaluated the virulence of *K. pneumoniae* in the zebrafish larvae, so we sought to develop an infection model suitable to generate pathological effects in the larvae, which could be quantified and compared among different bacterial strains, and also to evaluate the *in vivo* innate immune response of this host. In order to accomplish this, we tried two kinds of inoculation methods: static immersion and injection. Both have been successfully used to cause infections with different bacterial pathogens such as *Edwardsiella tarda* and *S*. Typhimurium (van Soest et al., 2011; Varas et al., 2017a). Regarding the injection method, we followed either a systemic infection or a localized infection approach, based on previous studies of bacterial pathogens using zebrafish embryos (Benard et al., 2012). Thus, attempting different routes of administration allowed us to set up a successful infection method, as well as to evaluate distinct host responses related to the bacterial entry mechanism, which can help discriminate between bacterial strains with diverse virulence potential.

### Systemic injection of different strains of *K. pneumoniae* into zebrafish larvae

**Figure 4:**
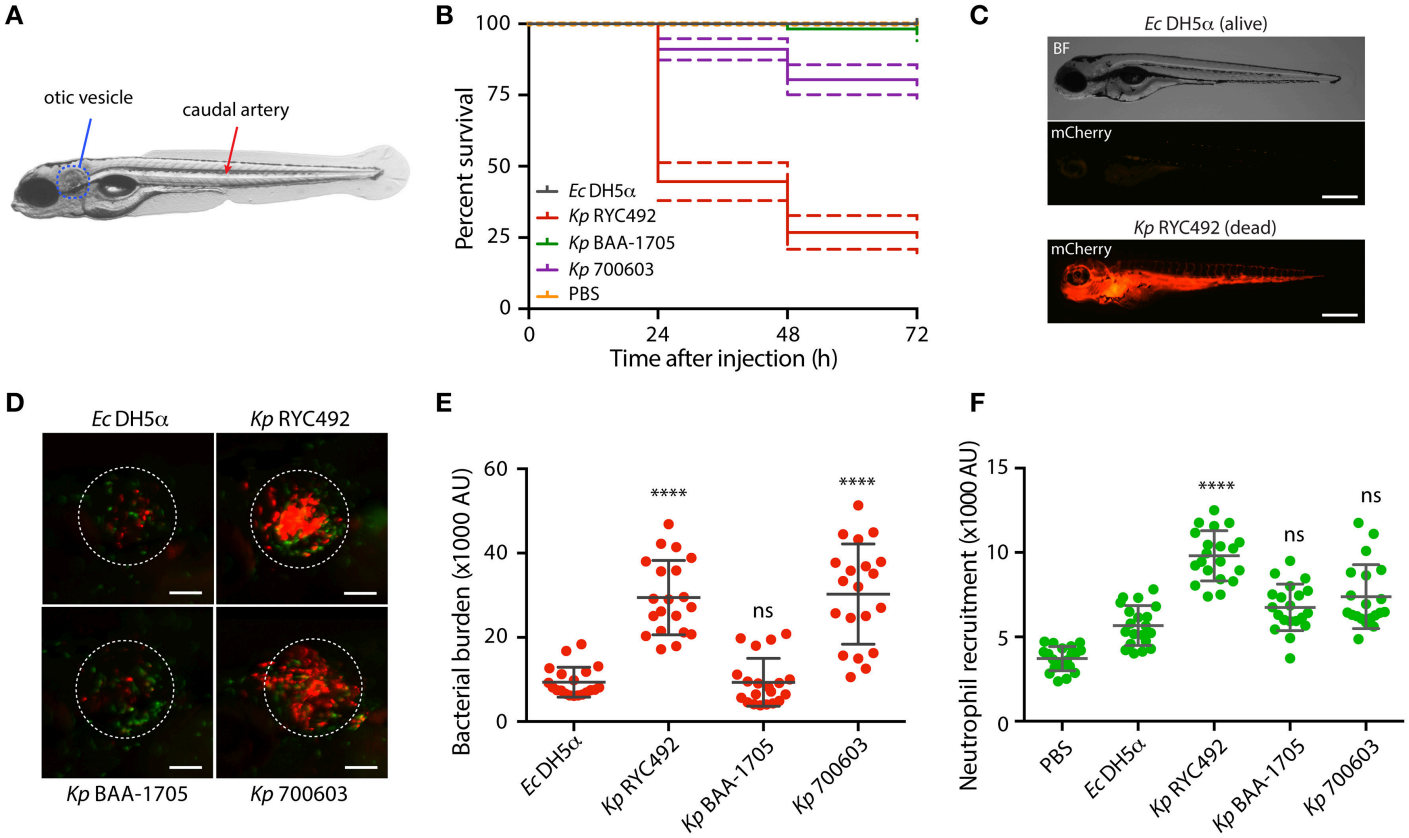
*K. pneumoniae* strains show different virulence behavior in the zebrafish larvae as revealed by survival curves and neutrophil recruitment. **(A)** Scheme of a 5-dpf zebrafish larva. Bacteria were injected in the dorsal caudal artery (red arrow) for establishing a rapid systemic infection, or in the otic vesicle (blue circle) for a localized infection. **(B)** Kaplan-Meier survival curves following injection of 3 dpf larvae with various strains of *K. pneumoniae* or with *E. coli* DH5α as a control. Larvae survival was monitored for 3 days after injection. The data are representative of three independent experiments, with a total of 57 larvae per bacterial strain. **(C)** Representative picture of a larva infected for 24 h with *K. pneumoniae* RYC492 strain, after acute bacteremia and death, and an unaffected larva injected with *E. coli* as a control. Bacteria expressing mCherry fluorescent protein is shown red. Scale bar, 500 μm. **(D)** Neutrophils attracted to the otic vesicle of Tg(*BACmpo:gfp*) zebrafish larvae injected with *K. pneumoniae* strains or *E. coli* DH5α after 24 h post-injection (hpi). Bacteria are red and neutrophils are green. The white dashed lines encircle the otic vesicle. Scale bar, 100 μm. **(E)** Bacterial burden in the otic vesicle of zebrafish larvae after 24 hpi. Measurements of red fluorescence from mCherry-labeled bacteria are plotted as arbitrary units (AU) for each *K. pneumoniae* strain or for *E. coli* DH5α. The data are representative of three replicates, with a total of 21 larvae per condition. **(F)** Neutrophils infiltrated in the otic vesicle of Tg(*BACmpo:gfp*) zebrafish larvae after 24 hpi. Measurements of green fluorescence from EGFP-labeled neutrophils are plotted as arbitrary units (AU) for larvae injected with *K. pneumoniae* strains, *E. coli* DH5α, or PBS. The data are representative of three independent experiments, with a total of 21 larvae per bacterial strain. Error bars represent the standard deviation among the replicates. Statistical differences compared to *E. coli* DH5α were assessed using a Kruskal-Wallis analysis followed by Dunn's multiple comparisons test. ^****^p < 0.0001.

To achieve a systemic infection, zebrafish larvae (3-dpf) were injected in the dorsal caudal aorta (Figure 4A) with 10,000 to 20,000 cells of mCherry-labeled *Kp* RYC492, *Kp* BAA-1705, *Kp* 700603, and *E. coli* DH5α (the latter as a non-pathogenic bacterial control previously validated in this host model; Varas et al., 2017a), or with PBS as an injection control. After daily monitoring of the injected larvae the survival was quantified and compared (Figure 4B). As expected, *E. coli* DH5α caused no lethality at any dose, condition, and time evaluated. Despite incubating the infected animals at 28°C (the optimal temperature for bacterial growth is 37°C), the larvae injected with *K. pneumoniae* strains showed an increased mortality over time. Zebrafish larvae injected with *Kp* BAA-1705 showed no significant lethality compared with the group injected with *E. coli* DH5α (Gehan-Breslow-Wilcoxon test, *p* = 0.155). On the other hand, *Kp* RYC492 caused the highest lethality, reaching 75% at 3 dpi, while *Kp* 700603 caused intermediate lethality, with 78.6% survival at the end of the experiment. Over time, larvae injected with *E. coli* cells completely cleared the infection, while severely affected fish infected with *K. pneumoniae* strains showed a marked bacteremia, spreading through the circulatory system and internal organs (Figure 4C; as control, a non-infected dead larva is shown in Supplementary Figure 4A). This occurred in all death events, regardless of the *K. pneumoniae* strain.

### Localized injection of different strains of *K. pneumoniae* into zebrafish larvae

The otic vesicle (Figure 4A) was selected as the larval tissue to inject the bacteria and generate a localized infection event. This is a confined compartment devoid of macrophages and neutrophils, and can be used to study the directed migration of innate immune cells (Benard et al., 2012). Bacterial cells or PBS (control) were carefully injected in the otic vesicle of Tg(*BACmpo:gfp*) zebrafish larvae harboring GFP-labeled neutrophils, and neutrophil recruitment was quantified by measuring green fluorescence inside the vesicle. Bacterial infection in this compartment normally causes neutrophil recruitment as soon as 3 h after injection (Benard et al., 2012). We observed a marked inflammation of the otic vesicle after 8 h of injection, which was similar between all subjects, even those exposed to *E. coli* DH5α (data not shown). To determine whether the fish immune cells are able to clear the bacteria and resolve, we examined the otic vesicle of injected fish 24 h after injection (Figure 4D; as control, a larva injected with sterile PBS is shown in Supplementary Figure 4B), when normally the clearance of non-pathogenic bacteria has occurred. The fluorescence of green-labeled neutrophils (Figure 4E) and red-labeled bacteria (Figure 4F) were quantified inside the otic vesicle from injected larvae. The bacterial burden was significantly higher in the larvae injected with *Kp* RYC492 and *Kp* 700603, compared to the larvae injected with *E. coli* DH5α (Dunn's multiple comparisons test, *p* < 0.0001). This correlates with the neutrophil recruitment, where a higher count was observed after injecting these two *Kp* strains, but only with statistical significance in the *Kp* RYC492 injected larvae (Dunn's multiple comparisons test, *p* < 0.0001). This result indicates that the *Kp* RYC492 strain (and *Kp* 700603 in a lesser extent) triggered a higher neutrophil recruitment in the injected area, and that these strains also show an increased ability to persist and/or replicate in the site of infection compared with *E. coli* DH5α or *Kp* BAA-1705.

### Bacterial infection of zebrafish larvae by static immersion

**Figure 5:**
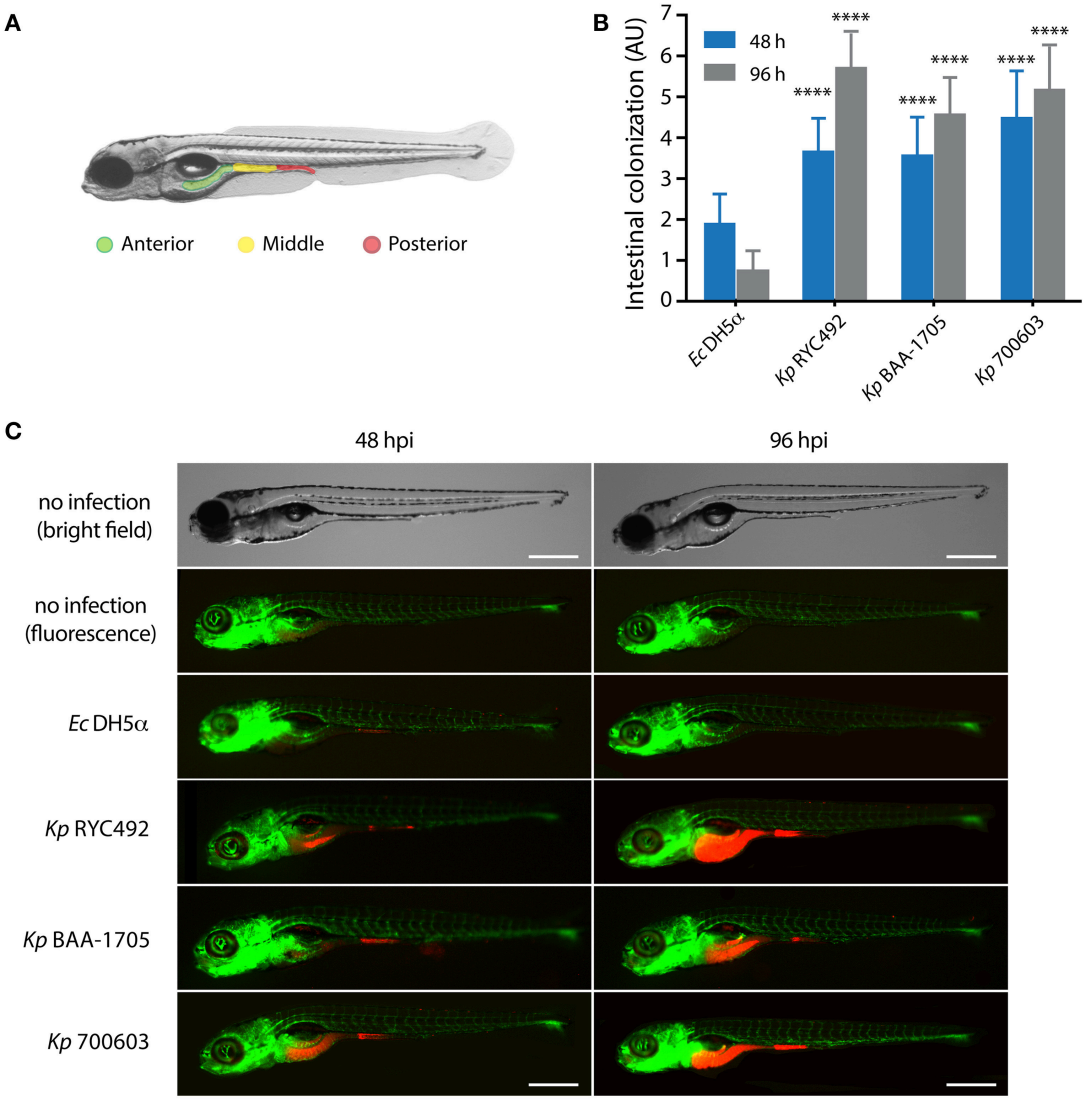
*K. pneumoniae* can infect zebrafish larvae by immersion and colonize its gastrointestinal tract. **(A)** Scheme of the gastrointestinal tract of a 5-dpf zebrafish larva, showing three arbitrarily defined zones (anterior, middle and posterior). **(B)** Semi-quantitative assessment of the intestinal colonization upon immersing zebrafish larvae in a suspension containing different *K. pneumoniae* strains or with *E. coli* DH5α. At the indicated time, larvae were anesthetized, observed under a fluorescence stereomicroscope, and the total intestinal colonization was scored in a scale from 1 to 9, following the criteria described in the Materials and Methods section. The data presented correspond to six replicates with a total of 75 larvae per bacterial strain. **(C)** Fluorescence microphotographs of zebrafish larvae representative of the data showed in **(B)**. Bright field images of representative non-infected larvae at 48 and 96 h post-immersion are presented for more clarity. The larvae vasculature is shown in green, while infecting bacteria are shown in red. Scale bar, 500 μm. ^****^p < 0.0001.

In order to explore whether *K. pneumoniae* can infect zebrafish through the natural entry route for this enteric pathogen, and based on previous studies (van Soest et al., 2011; Varas et al., 2017a,b), we adapted and optimized an immersion protocol for its use with this pathogen. The selected conditions included using Tg(*fli1:egfp*) zebrafish larvae (3 dpf), and co-incubating them with bacteria during a period of 48 h. This transgenic line has a GFP-labeled vasculature, and was chosen to better evaluate the occurrence of bacteremia after immersion, and to obtain a fluorescence signal from the whole larvae (green) to contrast the bacteria-derived fluorescence (red). Afterwards, the larvae were washed to remove unattached bacteria, and then monitored for 4 days after immersion (dpi). Washing was performed daily during the experiment. Unlike to that observed with injections into the circulatory system, no lethality was observed in larvae immersed in suspensions of any *K. pneumoniae* strain evaluated (data not shown). Since bacterial infections in zebrafish might cause consequences other than death, we monitored the fluorescence of the bacteria within larvae through live-cell imaging. Observation of the larvae under fluorescence revealed that the bacteria accumulated in the gastrointestinal tract. To evaluate and score the colonization, we artificially separated the gut into three major zones: anterior, middle and posterior (Figure 5A). The amount of fluorescence in each zone was observed and an intensity score was assigned: no colonization = 0, low colonization = 1, medium colonization = 2 and high colonization = 3. The sum of the values for each zone was calculated for each individual, representing the total intestinal colonization score (Figure 5B). Photographs of representative individuals of each condition are shown in Figure 5C. There were no clear differences between distinct *K. pneumoniae* strains when observing the pattern of colonized zones, although they appear to be prone to colonize the anterior and middle digestive tract rather than the posterior digestive system (Supplementary Table 1). The total intestinal colonization score determined for *E. coli* DH5α was significantly lower than that observed for all the *K. pneumoniae* strains assayed, both at 48 and 96 hpi (Two-way ANOVA, *p* < 0.0001). *E. coli* DH5α colonization significantly decreased at 96 hpi compared to 48 hpi, while the opposite was observed with the three *K. pneumoniae* strains, where an increased bacterial colonization was observed in a later time (Figures 5B,C).

In conclusion, while the immersion method allowed differentiating the non-pathogenic bacteria (*E. coli* DH5α) from the pathogenic bacteria (*K. pneumoniae* strains), the injection method allowed discriminating between strains from the same bacterial species with different degree of virulence (*Kp* RYC492 > *Kp* 700603 > *Kp* BAA-1705).

### *K. pneumoniae* RYC492 showed an increased biofilm formation and a thicker capsule

**Figure 6:**
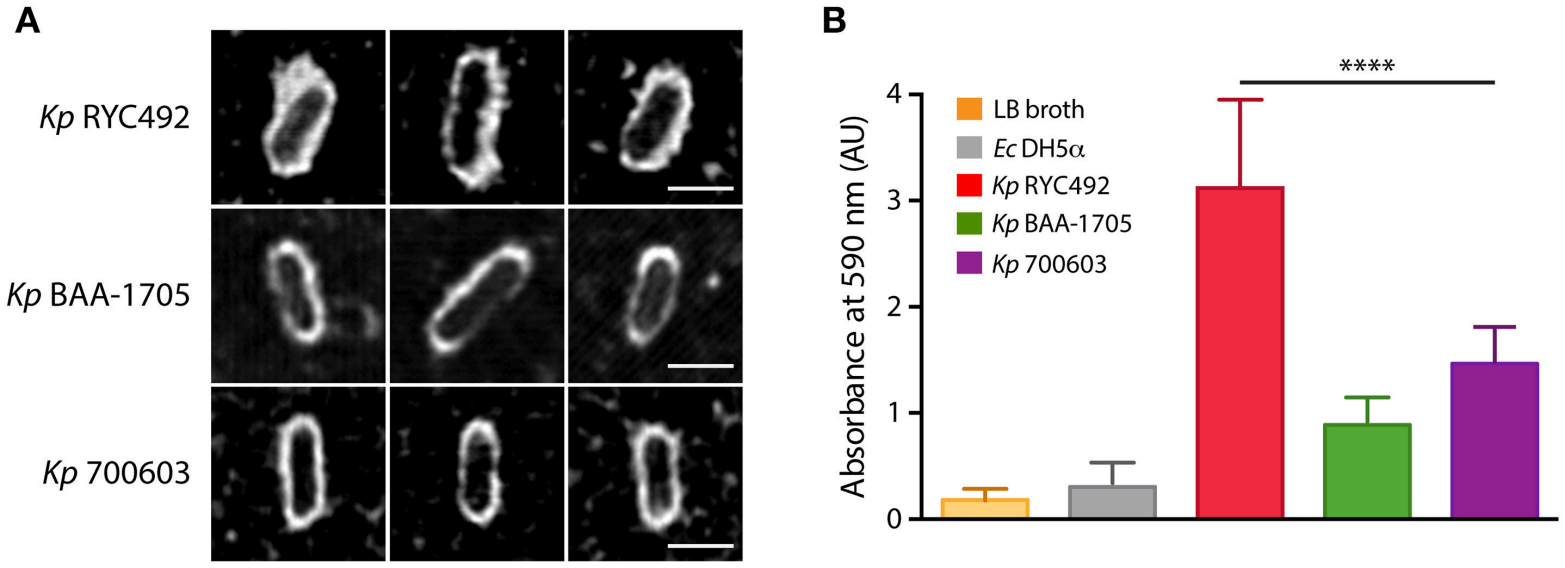
*Klebsiella pneumoniae* RYC492 virulence correlates with a more prominent and irregular capsule and with increased biofilm formation ability. **(A)** *K. pneumoniae* cells were subjected to classical capsule staining with India ink, and observed and photographed using confocal microscopy. Pictures of 3 representative cells of each bacterial strain are presented. The capsule corresponds to the white area between the bacillary cell and the black background. Scale bar, 2 μm. **(B)** Biofilm formation assessment of *K. pneumoniae* strains or *E. coli* DH5α upon growth in a 96-well plate during 18 h. Bacterial cells adhered to the wells were stained with crystal violet, where biofilm formation is proportional to the absorbance of the bound stain (measured at 590 nm). The values shown correspond to the mean of three independent experiments and the error bars represent the standard deviation. RYC492 strain showed a significantly higher ability to form biofilms than *Kp* 700603 (one-way ANOVA, ^****^p < 0.0001), and then the rest of the bacterial strains tested.

The assays presented above indicated that *Kp* RYC492 is virulent to both *D. discoideum* and zebrafish hosts, showing resistance to phagocytosis by amoeba cells, a great ability to colonize the zebrafish gastrointestinal tract, and to trigger a strong innate immune response in the otic vesicle injection model. In spite of the strong neutrophil recruitment unleashed, this immune response failed to reduce the bacterial burden in a normal time lapse, compared to non-pathogenic bacteria and to other *K. pneumoniae* strains. As described previously, the capsule is one of the most relevant virulence factors in this species, and indeed it has been previously related to resistance to phagocytosis, immunogenicity, biofilm formation, and tissue colonization. Furthermore, we observed that the macrocolonies formed on sheep-blood and LB agar plates by *Kp* RYC492 but not by *Kp* 700603 or *Kp* BAA-1705 showed a mucoid phenotype, which could also be related to the properties of its capsule. In view of this, we tested if *Kp* RYC492 possess the hypermucoviscous phenotype (highly associated with hypervirulent strains), which has been semi-quantitatively defined by a positive “string test.” This test is positive when a bacteriology inoculation loop is able to generate a viscous string of more than 5 mm in length by stretching bacterial colonies on an agar plate (Shon et al., 2013). However, after streaking and growing *Kp* RYC492 in sheep-blood agar and performing the test, it resulted negative, indicating that this strain is not hypermucoviscous (data not shown). Next, we sought to explore if a simple classical capsule staining using India ink could allow us to distinguish morphological peculiarities of the *Kp* RYC492 capsule compared with that of *Kp* 700603 and *Kp* BAA-1705. After staining, the samples were observed using confocal microscopy in a laser-mediated bright field mode, and several fields were photographed and analyzed. Figure 6A shows pictures of 3 representative capsule-stained individual cells from each strain. Additionally, an expanded set of pictures of this experiment is presented in Supplementary Figure 5, where stained cells were ordered by cell length to facilitate the comparison, and to allow appreciating the capsular morphology in all the variety of cell dimensions observed. While both *Kp* 700603 and *Kp* BAA-1705 strains showed a well-defined and regular round-shaped capsule, *Kp* RYC492 showed a notoriously thicker capsule, with an irregular morphology and a fuzzy aspect. Thus, the classical staining allowed us to easily differentiate the strains analyzed in terms of the capsular morphology, where a thicker and more irregular capsule in *Kp* RYC492 correlated to his more virulent phenotype.

To investigate if *Kp* RYC492 has an increased ability to form biofilms, we conducted a simple assay in 96-well plates to measure biofilm formation over an abiotic surface, previously used to the same end with other strains of *K. pneumoniae* (Bruchmann et al., 2015). As expected, *Kp* RYC492 showed a significantly higher ability to form biofilms than *E. coli* DH5α and then all other *K. pneumoniae* strains tested (Figure 6B). This correlates with its increased ability to colonize the zebrafish intestine, and with having a more prominent capsule.

### Genomic determinants of *K. pneumoniae* RYC492 virulence

In order to better understand the molecular basis of the differences observed among the *K. pneumoniae* strains in the multiple virulence phenotypes assessed, we analyzed the previously sequenced genome of *Kp* RYC492, *Kp* 700603, and *Kp* BAA-1705 in search for virulence factors, as well as to gain information regarding their determinants for capsule production. Since in a previous work we found that genomic islands (GIs) integrated into tRNA-coding genes (tDNAs) are an important source of virulence factors in this species (Marcoleta et al., 2016), we started identifying and characterizing the GIs present in the chromosome of the three *K. pneumoniae* strains (Table 2 and Supplementary Table 2). *Kp* RYC492 chromosome harbors 4 GIs from which GIE492, encoding the determinants for producing microcin E492 and salmochelin siderophore, has a suggested role in *K. pneumoniae* virulence (Marcoleta et al., 2016). No additional putative virulence factors were found in the rest of the GIs, except for a Fe^3+^/spermidine/putrescine transport system encoded in a GI integrated at a Ser tDNA. Besides, two GIs were identified in the *Kp* 700603 chromosome, corresponding to a prophage and a GI encoding many hypothetical proteins of unknown function. *Kp* BAA-1705 harbors five GIs in its chromosome. Three of them likely correspond to prophages, and the other two encoded many proteins of unknown function, none of them predicted as putative virulence factors. Thus, in agreement with its experimentally demonstrated virulence, only *Kp* RYC492 harbors putative pathogenicity islands integrated in its chromosome. Though, none of these factors could be clearly linked to phagocytosis resistance.

**Table 2:** Main genomic features of *K. pneumoniae* strains used in this study.

**Strain**	**Predicted VFs^a^**	**Probability of being a human pathogen^a^ (%)**	**Capsular genotype/serotype^b^**	**Genomic Islands^c^**
RYC492	294 (92)	88.7	*wzi*37; KL22 and KL37/K22 and K37	**-Asn tDNA:** GIE492 (~22 kbp). Encodes genes for production of salmochelin siderophore and microcin E492 (Marcoleta et al., 2016). **-Leu tDNA:** Unnamed (~5 kbp). Putative GI remnant. **-Phe tDNA:** Unnamed (~3 kbp). Putative GI remnant. **-Ser tDNA:** Unnamed (~19 kbp). Harbors genes related to c-diGMP signaling, a Fe^3+^/spermidine/putrescine transport system, and a phosphotransferase sugar uptake system.
BAA-1705	130 (52)	82.2	*wzi*154; KL107/Unknown	**-Arg tDNA:** Unnamed (~43 kbp). Putative prophage, encoding many proteins related to capsid and tail assembly. **-Leu tDNA:** Unnamed (~11 kbp). Putative prophage remnant. **-Thr tDNA:** Unnamed (~10 kbp). Putative prophage remnant. **-Asn tDNA:** Group II Asn tDNA-associated GI (~28 kbp; Marcoleta et al., 2016). Encodes proteins involved in conjugal transfer and many of unknown function. **-Phe tDNA:** Unnamed (~58 kbp). Encodes mostly hypothetical proteins of unknown function.
700603	50 (12)	84.2	*wzi*171; KL53/Unknown	**-Arg tDNA:** Unnamed (~47 kbp). Putative prophage, encoding many proteins related to capsid and tail assembly. **-Arg tDNA:** Unnamed (~47 kbp). Encodes many hypothetical proteins of unknown function.

a *The total number virulence factors and the probability of being a human pathogen were predicted using the PathogenFinder tool (Cosentino et al., 2013). The numbers in parenthesis indicate the amount of the predicted VFs corresponding to proteins of unknown function*.

b *Capsular genotype were determined using Kleborate (Wyres et al., 2016) and Kaptive (Lam et al., in review) tools, which compare the capsule biosynthesis genes found in a given K. pneumoniae genome with dedicated databases, and categorize them into defined wzi and KL groups. Only in the case of RYC492, the capsular genotype could be related to two putative K serotypes (K22 and K37)*.

c *For each genomic island, we indicated the tRNA coding gene (tDNA) in which it was found integrated, a previously given name (if available), its size (in kbp), and a brief description of the carried genes*.

Next, we analyzed and compared the whole genome sequences of the three *K. pneumoniae* strains using different bioinformatics tools. General and *K. pneumoniae*-specific identification of virulence factors were performed using PathogenFinder (Cosentino et al., 2013) and Kleborate (Lam et al., in review), respectively. *Kp* RYC492 showed the highest number of predicted virulence factors (294), although many of them correspond to proteins which function has not been addressed experimentally (Table 2, Supplementary Table 3). Despite *Kp* BAA-1705 and *Kp* 700603 showed a considerable smaller number of putative virulence factors (130 and 50, respectively) they were predicted as human pathogens by PathogenFinder with a probability of 82.2 and 84.2%, respectively, while KpRYC492 showed a score of 88.7%. Additionally, in the genome of *Kp* 700603 we could not find genes encoding putative homologs to proteins participating in type-III secretion systems (T3SS), such as those encoded in the *Salmonella* pathogenicity islands SPI-1 and SPI-2, which have a demonstrated role in invasion and intracellular survival, respectively (Que et al., 2013; Jennings et al., 2017). Regarding adhesion factors such as fimbriae, no differences where observed among the three strains. All of them harbor the *mrkA* and *mrkD* genes previously associated with adherence to animal tissues, as well as the *ecpRABCDE* operon, also associated to tissue's adherence (Alcántar-Curiel et al., 2013).

Kleborate and Kaptive tools were used to determine the capsular genotype of each strain, which in some cases can be associated to a defined capsular serotype (Wyres et al., 2016; Lam et al., in review). Kaptive tool classified the capsule *loci* of *Kp* BAA-1705 and *Kp* 700603 as KL107 and KL53, respectively, although none of them have been associated with determined serotypes. Kleborate tool indicated that *Kp* RYC492 possesses genes for the production of two capsular serotypes (K22 and K37). Unfortunately, to our knowledge there are no experimental data regarding the virulence and resistance to phagocytosis of other *K. pneumoniae* strains (besides RYC492) harboring any of these serotypes. Performing similar assays with additional strains of these serotypes will allow us to further test if there is an association between K22/K37 capsule types and an increased resistance to phagocytosis.

In conclusion and in agreement with the experimental data, genomic analyses allowed the identification of putative pathogenicity islands only in *Kp* RYC492, which also harbored the highest number of predicted virulence factors in different positions of its genome. Nevertheless, it was not possible to associate any of these factors with the phagocytosis resistance phenotype displayed by this strain. Regarding *Kp* 700603, no clear genetic determinants could be associated with its ability to replicate intracellularly in *D. discoideum*.

## Discussion

Until now, multidrug-resistant and hypervirulent *K. pneumoniae* strains have evolved separately in distinct clonal groups (Hennequin and Robin, 2016). However, given the high genomic plasticity of this species and that the dissemination of both resistance and virulence determinants were associated to similar mobile elements such as plasmids and genomic islands, it is likely that in the near future strains showing both traits will arise. This will certainly represent a challenge to global health, particularly since the interplay between resistance and virulence in this species is poorly understood. Despite its high clinical relevance, the lack of a good animal model for studying pathogenesis has hampered the virulence assessment of multiresistant *K. pneumoniae* strains, because in the available mice models they usually behave as avirulent (Diago-Navarro et al., 2014). Thus, new assays and the use of simple host models will contribute to the understanding of *K. pneumoniae* pathogenesis and antimicrobial resistance. In this context, we presented a series of assays to study *K. pneumoniae* virulence using the social amoeba *D. discoideum* and the fish *Danio rerio* as host models. These assays allowed us to observe striking differences in virulence-associated traits among distinct strains of this bacterial pathogen, including resistance to phagocytosis, invasiveness, intracellular survival, intestinal colonization, neutrophil recruitment and lethality. In each host model, these traits were monitored in different physiological contexts observing consistent results, supporting the conservation of the mechanisms involved in the interaction of *K. pneumoniae* with different hosts.

The use of non-mammalian host models for studying bacterial pathogenesis has allowed not only to facilitate the evaluation of virulence-associated phenotypes, but also to discover that many of the mechanisms required for infecting diverse hosts are essentially conserved, as well it is the molecular basis of the host's response to the infection. Thus, using different model hosts and exploiting the advantages that each of them offers, allows interesting comparisons regarding the virulence strategies and host defense mechanisms during the infection of phylogenetically distant organisms. In this work, we showed that *D. discoideum* and zebrafish larvae have several advantages to be used as models for study *K. pneumoniae* pathogenesis. Their optical transparency and the availability of *D. discoideum* mutant strains and transgenic zebrafish lines permitted to follow the infective process trough live-cell imaging and without requiring invasive sample acquisition. Also, their easy manipulation, rapid growth, and numerous progeny, allowed analyzing a numerous cohort and to perform several replicates, providing more statistical robustness to the conclusions. One of the limitations that have these models is that the infection cannot be performed at 37°C, in circumstances where such temperature is expected to be the optimum for the expression of several virulence factors of human pathogens. In this direction, previous studies demonstrated that more than 120 *E. coli* genes are overexpressed at 37°C, compared with that observed at 23°C, including several involved in the uptake and metabolism of amino acids, carbohydrates and iron (White-Ziegler et al., 2007). However, there is also evidence indicating that *Salmonella* Typhimurium exposed to sublethal heat stress (42°C) responds by altering the expression of several virulence factors, which further enhances the adhesion of bacterial cells to intestinal Caco-2 cells (Sirsat et al., 2011). Also, Yang et al. (2013) evaluated the expression of the *S*. Enteritidis virulence-related genes *spvR, hilA, sefA*, and *avrA* at different temperatures, showing that most of them are expressed significantly more at 42°C than a 37°C. Moreover, it was shown that *S*. Typhi can grow in seafood incubated at 22°C, and that key virulence-associated genes such as *invA* and *stn* were strongly expressed at this temperature (Kumar et al., 2015). Furthermore, in previous work we showed that relevant *S*. Typhimurium virulence factors required for infection of mammalian host cells and animal models at 37°C, including the type III secretion systems (T3SS) encoded in SPI-1 and SPI-2 pathogenicity islands, are also essential to invade and survive inside *D. discoideum* cells at 23°C (Riquelme et al., 2016). All this evidence points out that although mammalian's pathogens are indeed adapted to successfully infect these animal hosts at 37°C, they also can display a wide array of virulence traits at different environmental conditions. This is not surprising considering that part of the existence of many bacterial (human) pathogens occurs outside mammalian's body, where bacteria must interact and/or infect a variety of hosts in a changing environment.

As mentioned before, other alternative host models have been used to study bacterial pathogenesis, each of them showing advantages and limitations. One of the most used for studying *K. pneuminiae* pathogenesis is the larval state of the wax moth *G. mellonella* (Insua et al., 2013; Wand et al., 2013; Mills et al., 2017). This model can be maintained at 37°C, and thus allows to study the infective process at the same temperature than that occurring inside mammalian hosts. However, this host is not amenable for live-cell imaging, or for knocking-out host proteins, as *D. discoideum* and zebrafish are. Other cited benefit of using *G. mellonella* model is the multiple options for the delivery of pathogens, such as topical application, oral delivery and injection (Junqueira, 2012). However, this benefit is also valid for our host models, but with the difference that, in the case of zebrafish, we are using a vertebrate host where not only the innate inmune response but also the adaptive inmunity can be studied.

Phagocytes are a first line of defense against bacterial pathogens, playing a key role in the host's innate immune response to infection. The amoeba *D. discoideum* shares many traits with human phagocytes including the ability to ingest and kill bacteria, as well as key aspects of membrane trafficking, endocytic transport and sorting events (Bozzaro et al., 2008). Previous studies have explored the *Klebsiella*-*D. discoideum* interaction, identifying host genes required for the intracellular killing of *K. pneumoniae* (Benghezal et al., 2006). Remarkably, homologs of those genes also determined *Drosophila*'s susceptibility to this pathogen, supporting the conservation of *Klebsiella*-host interaction mechanisms across distant lineages. Our results are in line with this observation, since RYC492 resulted to be the most virulent *K. pneumoniae* strain for both the amoeba and zebrafish hosts. Moreover, the resistance to phagocytosis and the capsule properties seem to be key conserved determinants in the infection of different hosts. When we compared *Kp* RYC492 with the previously characterized *K. pneumoniae* MGH78578 strain, which was shown to be attenuated in a mice respiratory disease model and highly susceptible to phagocytosis by murine macrophages (Fodah et al., 2014), we also observed that *Kp* RYC492 was more virulent. Social development assays performed with *Kp* MGH78578 revealed that it behaved similar to *Kp* BAA1705 and *Kp* 700603, resulting to be mildly virulent to the amoeba (Supplementary Figure 6). This supports the utility of this model to compare the virulence of different *K. pneumoniae* strains. Unfortunately, the extreme resistance of *Kp* MGH78578 to most antibiotics prevented its transformation with a plasmid conferring red fluorescence required for most of the live-cell imaging experiments performed in this study.

Additional studies support the role of the capsule in *K. pneumoniae* virulence, where mutants in the biosynthesis of this structure are attenuated in a murine host model (Benghezal et al., 2006). Furthermore, Pan et al. (2011) found several capsular genes related to phagocytosis resistance in a *Dictyostelium* model, and that this trait could be linked to hypervirulence. Later, March et al. (2013) explored the role of *K. pneumoniae* surface structures on its interaction with *D. discoideum* and mouse alveolar macrophages (Broug-Holub et al., 1997). As expected, non-capsulated mutants were more susceptible to phagocytosis by both *D. discoideum* and the macrophages. Consistently, such mutants showed attenuated virulence when using a pneumonia mouse model (March et al., 2013). Similarly, we found that the production of a more prominent capsule correlated with an increased resistance to phagocytosis by *D. discoideum* and to a reduced clearance by immune host cells of zebrafish larvae, as observed for *Kp* RYC492.

Our assays using *D. discoideum* permitted the evaluation of additional aspects of *K. pneumoniae* virulence. Through exploiting the amoeba social development, we easily distinguished the highly virulent strain *Kp* RYC492 from the attenuated or mildly virulent strains *Kp* 700603 and *Kp* BAA-1705. Phagocytosis assays using vegetative amoeba cells allowed observing differences in the invasiveness among different strains, and to assess the strong resistance to phagocytosis displayed by *Kp* RYC492. These assays also revealed that *Kp* 700603 could replicate inside *D. discoideum* cells. Both resistance to phagocytosis and intracellular survival traits were confirmed by live-cell imaging of the *Klebsiella*-amoebae interaction. Recently, it was shown that a strain of *K. pneumoniae* could survive within macrophages in an intracellular compartment that did not fuse with lysosomes, named *Klebsiella* containing vacuole (KCV) (Cano et al., 2015). The evidence also indicated that *K. pneumoniae* has the potential to escape from the phagocyte. Our results support this observation. Detection by confocal microscopy of extracellular bacteria after 24 h post-infection in the gentamicin-protection assays strongly suggest that *Kp* 700603 could escape from *D. discoideum* cells upon replication. It has been shown that *K. pneumoniae* capsule is dispensable for intracellular survival and that it would be down regulated inside the KCV (Cano et al., 2015). Accordingly, we observed no positive correlation between capsule properties and intracellular survival, where the strain showing the most prominent capsule (*Kp* RYC492) was unable to replicate inside amoeba cells. The assays described here could be used as a basis to further investigate the ability of certain *K. pneumoniae* isolates to survive inside phagocytic cells. A variety of *D. discoideum* strains harboring fluorescent fusions of proteins localizing to different subcellular compartments are available from Dicty Stock center. Fluorescent labeling of distinct structures of the endocytic pathway would allow to track *in vivo* the fate of *K. pneumoniae* cells upon host cell invasion, and to identify key events that determine their survival or killing. Furthermore, *D. discoideum* has been used as a model to study autophagy and its role in controlling infection when invasive bacteria escape from endosomal compartments to the cytosol (Calvo-Garrido et al., 2010). *D. discoideum* strains mutant in key autophagy proteins can be used to assess the role of this process in the response of the host to *K. pneumoniae* intracellular survival. Moreover, the simple manipulation and rapid growth of this amoeba allows to set up simple assays such as those proposed herein to evaluate a large number of *K. pneumoniae* strains or mutants, in the search for genes linked to intracellular survival.

To our knowledge, this is the first report using zebrafish larvae to study *K. pneumoniae* virulence. A recent work used adult zebrafish to test the safety and the efficacy *in vivo* of *Streptomyces*-derived molecules with antibacterial activity against multiresistant *K. pneumoniae* (Cheepurupalli et al., 2017). An intramuscular injection protocol was optimized to produce clinical and sub-clinical infections with this pathogen, and to successfully test the efficacy of the antimicrobials *in vivo*. This points out the usefulness of zebrafish model to test *in vivo* molecules directed to counteract *K. pneumoniae* infections. Our results showed that this model could also be used to study *K. pneumoniae* virulence and the associated host's immune response. Different infection methods (injection/immersion) allowed evaluating traits such as lethality, neutrophil recruitment, bacterial clearance, and intestinal colonization. Remarkably, immersion assays showed that zebrafish larvae could be easily infected with *K. pneumoniae* through its natural route of entry, leading to the colonization of different zones of the gastrointestinal tract. Noteworthy, Cheepurupalli et al. (2017) observed that immersion in doses of *K. pneumoniae* up to 10^12^ CFU/mL did not lead to clinical symptoms of adult fish, which only were achieved after intramuscular inoculation of 10^12^ CFU. In our assays, infection by immersion was achieved using 5–8 10^8^ CFU/mL, while 15,000–18,000 CFU were used for injections. This points to an increased susceptibility of the larvae compared to adult zebrafish as a key element to reach *K. pneumoniae* infections through immersion. Additionally, using early stages of the zebrafish development allowed us to take advantage of the transparency of the larvae for live-cell imaging studies and to evaluate a high number of individuals for robust statistics analyses. Due to the small size of the larvae, the infections could be performed in 6-well plates, reducing the space requirements and simplifying the manipulation of the animals. On the other hand, Cheepurupalli et al. (2017) used *ex vivo* cultures of zebrafish liver to evaluate histopathology and hepatotoxicity of the antimicrobials tested. Unfortunately, such evaluations were not performed after bacterial infection. It would have been interesting to see if *K. pneumoniae* can recapitulate a liver abscess in adult zebrafish, and thus using similar *ex vivo* liver cultures to study this kind of infection. Our assays using zebrafish larvae indicated that *Kp* RYC492 showed the highest lethality when injected into the bloodstream, as well as the highest neutrophil recruitment and bacterial burden after 24 h of being injected into the otic vesicle, a compartment normally devoid of neutrophils. This kind of immune cells is crucial for early-stage defense against *K. pneumoniae* in mouse infection models (Fukutome et al., 1980; Rehm et al., 1980). A recent study showed that a K1 clinical isolate could resist neutrophil-mediated killing remaining viable up to 24 h after infection, whereas infection of its acapsular mutant was cleared within 12 h (Lee C. H. et al., 2017). Also, in another report it was demonstrated that there is a good correlation between capsule type (mainly serotypes K1 and K2) and resistance to both extracellular and phagocytic killing by neutrophils isolated from human patients (Lee I. R. et al., 2017). Our results indicated that the higher neutrophil recruitment and the reduced clearance displayed by *Kp* RYC492 in the zebrafish model, correlated with its strong resistance to phagocytosis by *D. discoideum*, and with the production of a more prominent and irregular capsule.

Besides the capsule, other factors such as production of siderophores or antibacterial toxins are likely to contribute to *K. pneumoniae* virulence. In a previous study, we found that the GIE492 island, coding for the production of microcin E492 and salmochelin, was highly prevalent among liver abscess-associated *K. pneumoniae* (Marcoleta et al., 2016). Although not yet experimentally demonstrated, MccE492 production would permit the prevalence of the producers over surrounding cells competing for the same siderophores and thus increasing their iron supply. This effect would be potentiated by the co-production of salmochelin siderophore. Also, it is possible that MccE492 amyloid plays a role in extracellular matrix synthesis and biofilm formation, as observed for the curli fibers produced by *Escherichia* and *Salmonella* (Chapman et al., 2002; Wang et al., 2006) and the TasA protein from *Bacillus subtilis* (Romero et al., 2010). The direct assessment of these hypotheses has been hampered by the impossibility of making chromosomal deletions in the *Kp* RYC492 strain, although studies using alternative approaches are currently being performed (Marcoleta et al., unpublished results). Regarding salmochelin production, a previous work with hypervirulent *K. pneumoniae* showed that aerobactin but not yersiniabactin, salmochelin, or enterobactin, were required for virulence over *in vivo* infection models (Russo et al., 2015). However, additional studies are required to understand the specific role of the wide repertoire of siderophore production systems that compose the pangenome of this species. Further supporting the increased virulence of *Kp* RYC492, we showed that this strain carries a second putative pathogenicity island encoding a phosphotransferase system for the uptake of sugars and a Fe^+3^/spermidine/putrescine transport system, this last related to virulence in several pathogens (Shah and Swiatlo, 2008). Also, its genome harbors the highest number of predicted virulence factors compared with *Kp* 700603 and *Kp* BAA-1705 (Supplementary Table 3). Unfortunately, most of these genes code for proteins of unknown function.

In conclusion, our results showed that the surrogate host models *D. discoideum* and zebrafish larvae offer several advantages for the study of *K. pneumoniae* virulence. Moreover, these models seem to be very promising tools in the screening for novel anti-virulence molecules against multi-drug resistant pathogens including *K. pneumoniae*. This was successfully accomplished using *D. discoideum* as a host model to test both the efficacy and the safety of molecules with anti-virulence potential over *Pseudomonas aeruginosa* (Bravo-Toncio et al., 2016).

## Author contributions

AM, RL, and MV conceived the work. AM, MV, JO-S, FC, MA, CS, OM, and RL designed the experiments, analyzed the data and interpreted the results. AM, MV, LV, JO-S, CB-P, and AS conducted the experiments. AM, MV, JO-S, and RL wrote the manuscript. AM, MV, JO-S, LV, CB-P, FC, MA, CS, OM, and RL critically revised the manuscript. All the authors approved the final version of the manuscript.

### Conflict of interest statement

The authors declare that the research was conducted in the absence of any commercial or financial relationships that could be construed as a potential conflict of interest. The reviewer SD and handling Editor declared their shared affiliation.
