# Supplementary material for: Evaluating Different Virulence Traits of Klebsiella pneumoniae Using Dictyostelium discoideum and Zebrafish Larvae as Host Models
Source: Front Cell Infect Microbiol. 2018 Feb 9;8:30. doi: 10.3389/fcimb.2018.00030 (PMC5811510; doi:10.3389/fcimb.2018.00030)
Supplement: Supplementary file 1 [file Table1.pdf]

SUPPLEMENTARY MATERIAL

Evaluating different virulence traits of *Klebsiella pneumoniae* using *Dictyostelium discoideum* and zebrafish larvae as host models.

Andrés E. Marcoleta[1,*,+], Macarena A. Varas[1,+], Javiera Ortiz-Severín[3], Leonardo Vásquez[1], Camilo Berríos-Pastén[1], Andrea Sabag[2], Francisco P. Chávez[3], Miguel L. Allende[4], Carlos Santiviago[2], Octavio Monasterio[1], Rosalba Lagos[1,*].

Correspondence: Rosalba Lagos: rolagos@uchile.cl
Andrés E. Marcoleta: amarcoleta@uchile.cl

Supplementary Table 1. Intestinal colonization score for each of the defined intestinal segments at 48 and 96 h post immersion (hpi).

	K. pneumoniae ATCC BAA-1705			*K. pneumoniae* ATCC 700603			*K. pneumoniae* RYC492			*E. coli* DH5α		
	**A**	**M**	**P**	**A**	**M**	**P**	**A**	**M**	**P**	**A**	**M**	**P**
48 hpi	1.76	1.85	1.30	2.59	2.04	1.55	1.72	1.89	1.39	1.27	1.32	1.07
96 hpi	2.23	2.08	1.45	2.81	2.53	1.59	2.26	2.54	1.47	0.30	0.69	0.50

For each strain, the mean score of the cohort is shown for the three arbitrarily intestine segments defined, A: anterior, M: middle, P: posterior

Supplementary Tables 2 and 3 are provided as two separate spreadsheets:

Supplementary Table 2. Genomic Islands Identified in *K. pneumoniae* RYC492, ATCC BAA-1705 and ATCC 700603 (Supplementary Table 2.xls).

Supplementary Table 3. Predicted virulence factors identified in the genomes of *K. pneumoniae* RYC492, ATCC BAA-1705 and ATCC 700603, using the tool PathogenFinder (Supplementary Table 3.xls)

A

Non inoculated

Kp RYC492

Kp 700603

Kp BAA-1705

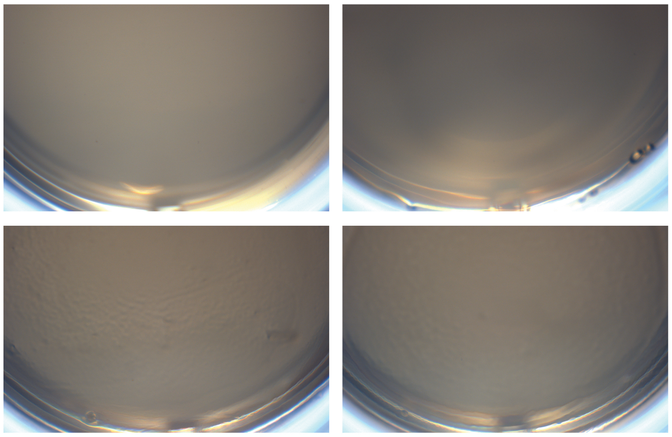

B

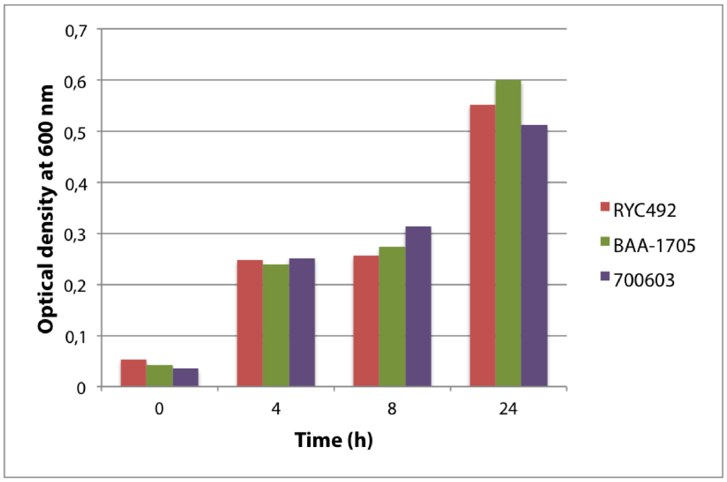

Supplementary Figure 1. *K. pneumoniae* strains RYC492, 700603 and BAA-1705 show similar growth at 23ºC in liquid and solid N medium. (A) 30 μL of overnight cultures of each strain were seeded into plates containing N agar, and incubated overnight at 23 ºC. Then, the surface of the agar was photographed, showing that all the strains tested presented a similar growth in the mentioned conditions, generating a thin lawn of bacteria in all the cases. (B) Overnight cultures of each strain were used to inoculate flasks containing N broth (dilution 1/100). Then, the cultures were incubated at 23ºC and the bacterial cell growth was monitored over time following the optical density at 600 nm.

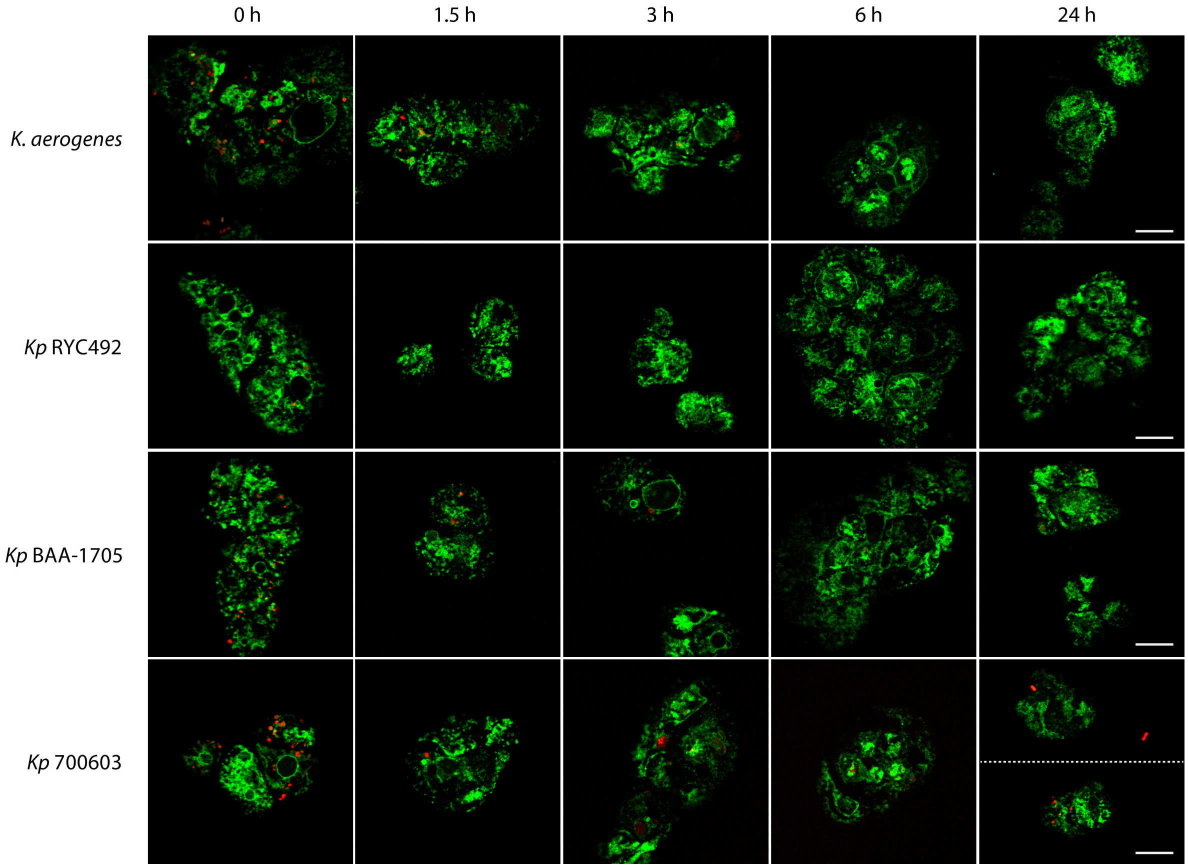

Supplementary Figure 2. Confocal microscopy images of the assays to evaluate intracellular survival of *K. pneumoniae* using *D. discoideum* as host cell model, as showed in Figure 2 but without the bright field channel. *D. discoideum* cells are green and bacterial cells are red. Scale bar, 10 µm.

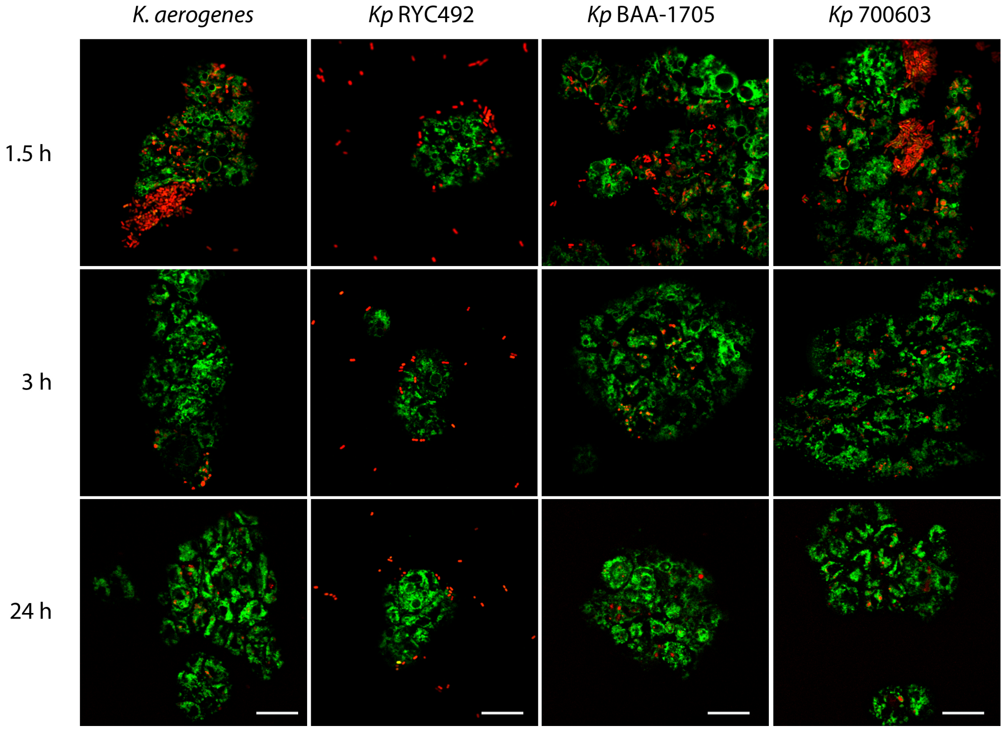

Supplementary Figure 3. Confocal microscopy images of the assays to evaluate resistance to phagocytosis of *K. pneumoniae* using *D. discoideum* as host cell model, as showed in Figure 3 but without the bright field channel. *D. discoideum* cells are green and bacterial cells are red. Scale bar, 10 µm.

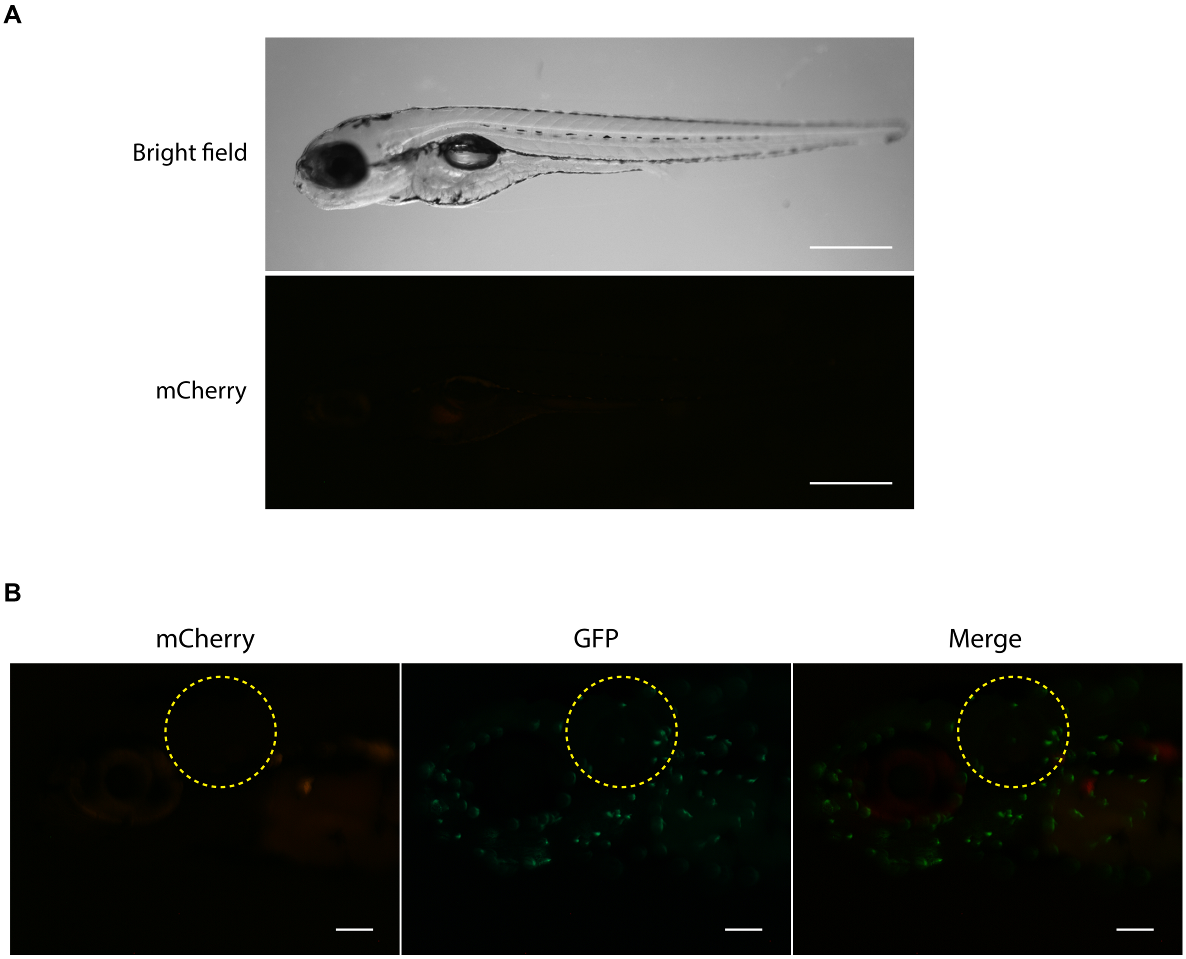

Supplementary Figure 4. (A) Non-infected zebrafish larvae euthanatized by anesthesia overdose showed little or no red fluorescence, in contrast to larvae dead by bacteremia upon infection with *Kp* RYC492 (as shown in Figure 4). Scale bar, 500 μm. (B) Larvae injected into the otic vesicle with sterile PBS (control condition) showed no red fluorescence in this area when evaluated 24 h post infection. The encircled area corresponds to the otic vesicle. Scale bar, 100 μm.

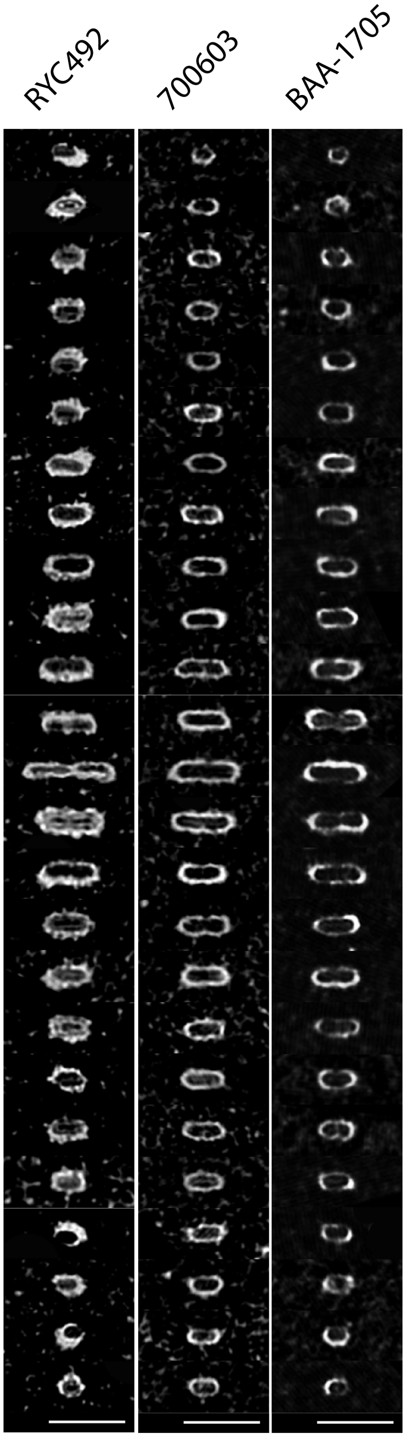

Supplementary Figure 5. Capsular morphology of *K. pneumoniae* strains RYC492, BAA-1705 and 700603. Cells were subjected to classical capsule staining with India ink and observed and photographed using confocal microscopy. Pictures of 25 representative cells of each bacterial strain are presented. Individual pictures were ordered by cell length to facilitate the comparison, and to allow appreciating the capsular morphology in all the variety of cell dimensions observed. The capsule corresponds to the white area between the bacillary cell and the black background. Scale bar, 5 µm.

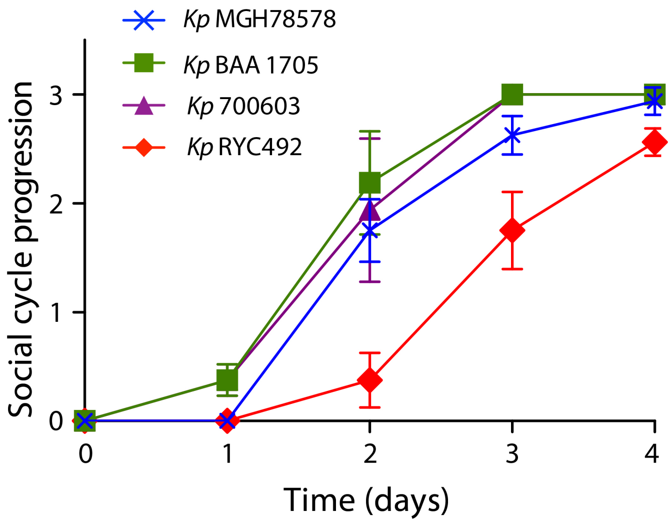

Supplementary Figure 6. Social development assay using *D. discoideum* to compare the virulence between the previously characterized strain *K. pneumoniae* MGH78578, and the *K. pneumoniae* strains characterized in this study (RYC492, 700603 and BAA-1705). Semi-quantitative assessment of the social cycle progression in presence of different *Klebsiella* strains using several replicates. The symbols represent the mean of three independent experiments and the error bars show the standard deviation. The details on scoring criteria can be found in the Materials and Methods section.
